# The emergence and adaptive use of prestige in an online social learning task

**DOI:** 10.1038/s41598-020-68982-4

**Published:** 2020-07-21

**Authors:** C. O. Brand, S. Heap, T. J. H. Morgan, A. Mesoudi

**Affiliations:** 1 Human Behaviour and Cultural Evolution Group, Department of Biosciences, College of Life and Environmental Sciences, University of Exeter’s Cornwall Campus, Penryn, UK; 2 Department of Biological and Environmental Science, University of Jyväskylä, Jyvaskyla, Finland; 3 School of Human Evolution and Social Change, Arizona State University, Tempe, USA; 4 Institute of Human Origins, Arizona State University, Tempe, USA

**Keywords:** Cultural evolution, Human behaviour

## Abstract

Prestige-biased social learning occurs when individuals preferentially learn from others who are highly respected, admired, copied, or attended to in their group. This form of social learning is argued to reflect novel forms of social hierarchy in human societies, and, by providing an efficient short-cut to acquiring adaptive information, underpin the cumulative cultural evolution that has contributed to our species’ ecological success. Despite these potentially important consequences, little empirical work to date has tested the basic predictions of prestige-biased social learning. Here we provide evidence supporting the key predictions that prestige-biased social learning is used when it constitutes an indirect cue of success, and when success-biased social learning is unavailable. We ran an online experiment (n = 269) in which participants could copy each other in real-time to score points on a general-knowledge quiz. Our implementation of ‘prestige’ was the number of times someone had previously been copied by others. Importantly, prestige was an emergent property of participants’ behaviour during the experiment; no deception or manipulation of prestige was employed at any time. We found that, as predicted, participants used prestige-biased social learning when the prestige cue was an indirect cue of success, and when direct success information was unavailable. This highlights how people flexibly and adaptively employ social learning strategies based on the reliability of the information that such strategies provide.

## Introduction

Prestige-biased social learning occurs when individuals preferentially learn from others who are highly respected, admired, copied, or attended to in their group^1,2^. This social learning bias is argued to reflect novel forms of social hierarchy in human societies^1^, and, by providing an efficient short-cut to acquiring adaptive information, to underpin the cumulative cultural evolution that has contributed to our species’ ecological success^3,4^. Social learning in general allows humans to acquire vast amounts of adaptive information from others, from acquiring detailed tool-making skills face-to-face, to online information exchange that pervades society today. However, for social learning to be adaptive, people need to be selective in who they copy, and extensive research in the last few decades has identified particular learning biases that facilitate the efficient acquisition of relevant knowledge and skills^5–8^. These include success bias, where people preferentially copy successful individuals, and prestige bias, where people use indirect cues of success to preferentially copy the most prestigious individual in the population.

Prestige as an indirect cue to success was first suggested by Boyd and Richerson^9^ and further developed by Henrich and Gil-White^1^. The latter authors proposed that, given variation across potential demonstrators in skill and/or knowledge, the tendency to copy the most skilled or knowledgeable demonstrator (known as success-biased or payoff-biased transmission) would create a market for copying opportunities in which people ingratiate themselves to highly skilled/knowledgeable people in exchange for access to their skills/knowledge. Thus, skilled/knowledgeable people are frequently copied by others, receiving widespread admiration, respect, or deference, which, according to Henrich and Gil-White’s definition, constitute “prestige”. Consequently, rather than evaluating the success of multiple possible models, it may be less costly, and therefore adaptive, for people to use prestige as a short-cut to identify who to copy. Reliable indirect cues might be ‘being copied’, ‘being paid attention’, ‘being admired’ or ‘being freely given material goods or social privileges’.

Henrich and Gil-White^1^ argued that this use of prestige bias relies on novel forms of social hierarchy in human societies where skilled/knowledgeable people attain high social rank and status. This prestige hierarchy is independent of dominance hierarchies which are shared with other social species, and in which individuals acquire rank via coercion or resource control. Prestige bias can also facilitate human cumulative cultural evolution where beneficial traits are accumulated over time, and which arguably underpins our unique ecological success^3,4^. Models show that cumulative culture crucially depends on some form of selective success bias^10,11^. Following the above logic, prestige bias, by acting as an effective short-cut to success bias, is a potentially important mechanism for cumulative cultural evolution.

Subsequent research has tested some of the assumptions and consequences of Henrich and Gil-White’s theory of prestige bias^2^. Ethnographic studies of small-scale societies have shown links between prestige (or proxies of prestige such as respect) and knowledge or skill in domains such as hunting^12,13^. However, such evidence is mixed: other studies have found no link between ethnobotanical knowledge and prestige^14^, and prestige is mentioned surprisingly infrequently across the ethnographic record in the context of skill or knowledge acquisition^12^. Furthermore, these studies did not directly measure or manipulate prestige-biased social learning, but looked at population-level correlations between prestige cues and knowledge/skill. Similarly, studies of industrialised populations have found links between prestige as measured via questionnaire scales and knowledge/skill in lab tasks^15,16^, but not examined whether such links emerge due to the social learning dynamics hypothesised by Henrich and Gil-White.

Some studies have examined prestige biased social learning directly, but again with mixed findings. Chudek et al.^17^ found that Canadian children were more likely to copy the preferences of an adult demonstrator to whom bystanders had been preferentially attending, rather than a demonstrator who had been ignored. However, a second study with a larger sample size found that children were no more likely to copy irrelevant actions performed by a high prestige demonstrator than a low prestige demonstrator. Atkisson et al.^18^ found that adult participants used indirect prestige cues, specifically how much time other participants had been viewed within a group, when choosing from whom to copy in an artifact-design computer task. Surprisingly, these indirect prestige cues were used just as frequently as direct success information (score in the task), despite the viewing times being fictional and therefore unreliable. This in fact goes against Henrich and Gil-White’s theory, which predicts that indirect prestige cues should only be used when direct success cues are unavailable, and when the indirect prestige cues provide some indication of, or correlation with success. However, the design of Atkisson et al.^18^ has several limitations, such as only presenting success information in the final set of trials, potentially leading to over-training on prestige cues^2^.

Our aim here is to fill this gap in the literature and provide a systematic test of the basic assumptions underlying the theory of prestige biased social learning. We test two key hypotheses which stem from Henrich and Gil-White’s theory that prestige biased social learning involves the use of indirect cues of success to select demonstrators from whom to learn. Defining prestige bias as preferentially copying demonstrators who have been copied the most by others, we hypothesise that:

1. Prestige bias is only adaptive, and thus only employed, when previous copiers had access to demonstrators’ success information, and not when previous copiers only had access to irrelevant information.
2. Prestige bias is only adaptive, and thus only employed, when direct access to demonstrators’ success is unavailable; otherwise direct cues of success will be preferred.

We test these hypotheses in a live, online, knowledge-based task. Participants answered binary-choice quiz questions based on four different categories of knowledge. They could either answer directly, or choose to copy the answer of another group member in real-time. Importantly, prestige was defined as the number of times a participant was copied by others; no deception or manipulation of prestige cues was employed at any time. We manipulated access to prestige information, and the quality of prestige information, across three different conditions. Across all conditions, prestige information consisted of the number of times potential demonstrators had been copied by others in the first round of the quiz. In the Control Condition, this prestige information was irrelevant and unrelated to success information: it was generated from participants copying each other on the basis of player ID numbers that were randomly generated. In the Prestige Condition, prestige information was relevant in that it was generated from participants copying each other on the basis of success information (score during the first round of the quiz). In the Success Condition, prestige information was relevant as in the previous condition, but direct success information was also available alongside it. We found, as predicted by our two hypotheses, that prestige information was preferentially used in the Prestige Condition, but not in the Control Condition or the Success Condition. Our study provides evidence that prestige biased social learning operates in a live, online, knowledge-based task where participants can freely choose if, when, and from whom they want to copy.

## Methods

Here we detail an online social learning experiment that was programmed via the open source experimental automation software ‘Dallinger’ which recruits participants via Mechanical Turk. We also conducted two prior studies that informed the methodological details of this study; one via Qualtrics with a UK sample, and one via an abstract social learning game based in Finland. For detailed information on the methods and results of these previous studies, please see the supplementary material. We discuss the similarities and differences of the results of all three studies in the Discussion section of this paper. All methods were carried out in accordance with relevant guidelines and regulations and granted ethical approval by the University of Exeter Biosciences ethics committee.

### Participants

We aimed to recruit ten groups of ten participants via Mechanical Turk for each of the three conditions, giving 300 participants altogether. However some participants dropped out of their session during the instructions/practice round, giving a starting sample size of 269, with 225 participants reaching the final demographics page. Nonetheless, we maintained a minimum of 5 participants in each networked group, with 16 of the 30 groups maintaining a full complement of 10 participants throughout. All participants were above the age of 18 (age range 19–71, mean age = 35.8), with 159 men and 119 women. All were given a monetary reward for their time of USD$10, and had the opportunity of winning a bonus payment of $20 if they scored over 85% in the quiz. 16 participants received the $20 bonus payment. All participants provided informed consent before being able to proceed to the task. The consent form informed them that their participation was entirely voluntary, their data were entirely anonymous, and they could withdraw their involvement at any time by closing their browser. It is worth noting that, although we had 44 participants drop out of the experiment, we found no evidence of selective attrition, in that there were no behavioural differences between those who dropped out (up until the point in which they dropped out) and the remaining participants, in terms of average score, copying rate, and answering questions in time. Over half of the drop-outs (24/44) were during the instructions for Round 2 which suggests these participants were automatically eliminated by our experiment. This was a feature of the experiment; if any participant took over two minutes to answer the comprehension check for the Round 2 instructions, they were eliminated from the group so as to allow the rest of their group to continue. This may be because participants either got distracted, were not paying attention, or had an internet problem. Participants were able to contact us to claim reimbursement if they felt they had been unfairly eliminated at this stage.

### Materials

**Figure 1:**
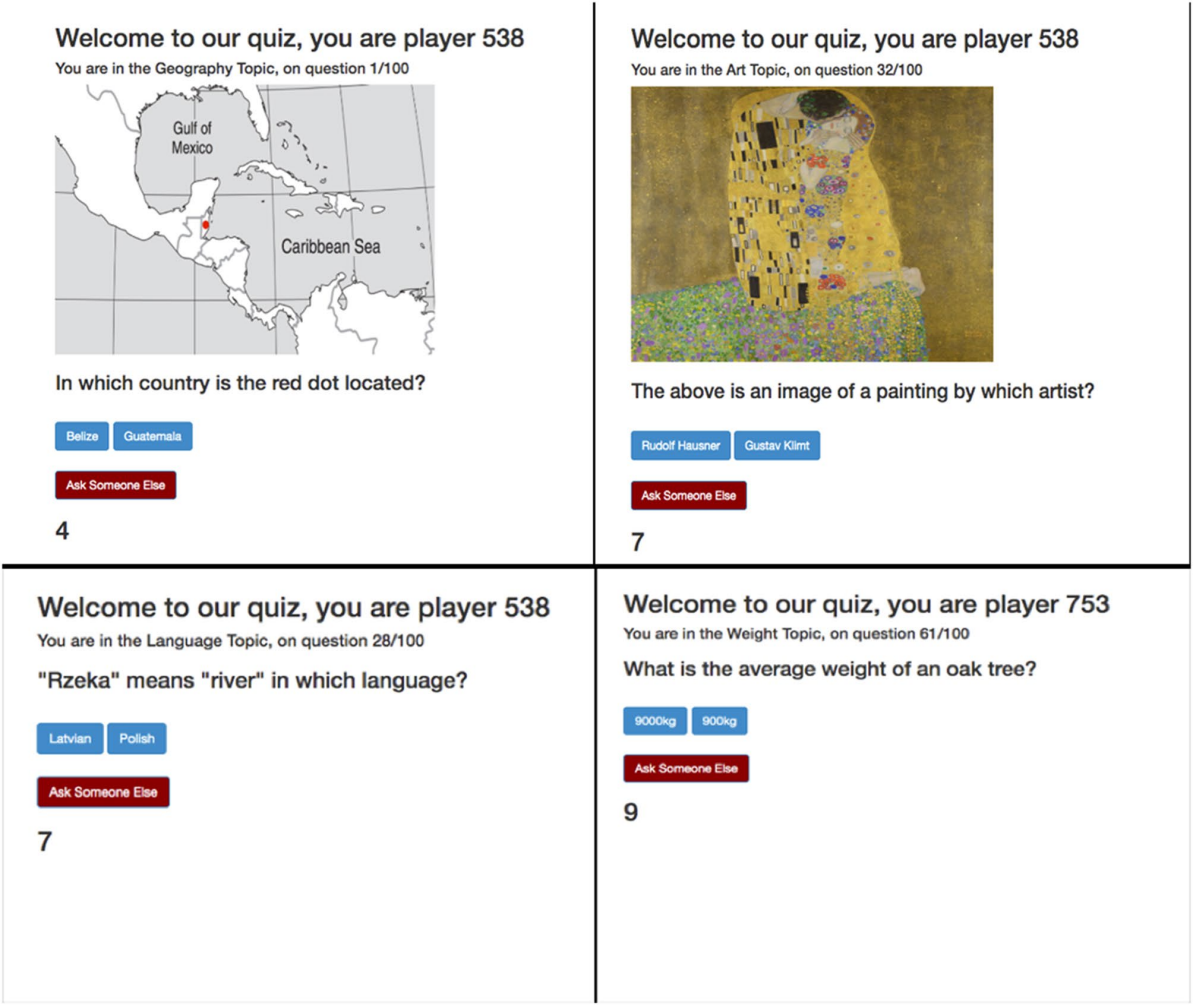
Four example screenshots representing questions from the Geography, Art, Language and Weight topics as seen by participants. Half the questions from each topic included images and half did not. Participants could either click one of the two blue buttons showing two possible answers (one correct, one incorrect), or click the red button which allows participants to copy someone else within their group. The number at the bottom is a countdown timer that forces participants to answer within 15 s. Player ID numbers are generated randomly for each participant when they begin the quiz. Here, three example screenshots come from one player (ID 538) and one from another player (ID 753). In reality, all participants view each question simultaneously. Note: all map images used in the quiz were sourced from https://www.eduplace.com/ss/maps/ and edited accordingly.

The open source experimental automation platform Dallinger (https://docs.dallinger.io/en/latest/) was used to generate a game in which groups of players could play and interact simultaneously. Participants answered 100 questions with two alternative answers each, one correct and one incorrect. The 100 questions were split into four categories of 25 questions each: “World Geography”, “Weight Estimation”, “Language Recognition”, and “Art History”. An example question for each category is given in Fig. 1. These questions were designed to potentially reflect real life experiences, rather than be a measure of formal educational attainment. For example, the Weight Estimation topic asked participants to estimate the heavier of two items, which may be learned as part of various jobs or hobbies (e.g. by engineers, health workers, mechanics, weight-lifters, bakers etc.).

### Procedure

Participants were assigned to a group of ten on starting the game. Once the group had ten individuals who had completed the consent form and read the instructions, the game began.

**Figure 2:**
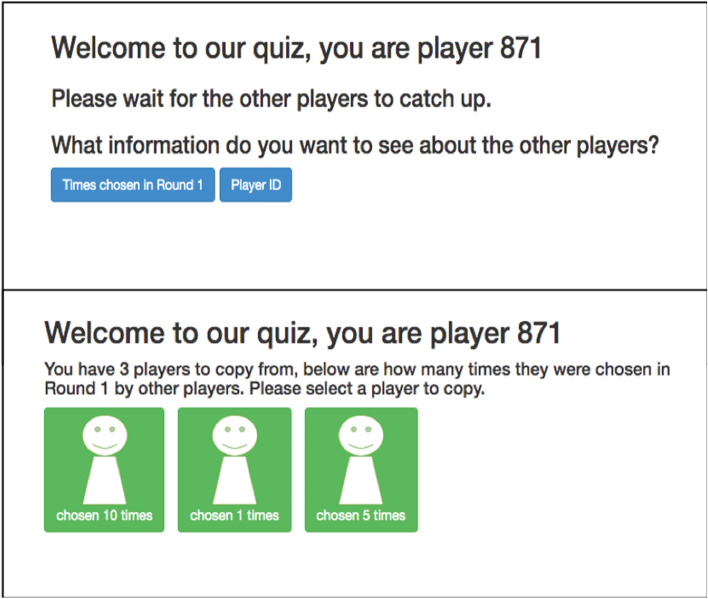
An example screenshot representing the choice a participant is given after selecting “Ask Someone Else” in Round 2 of the Prestige Condition and Control Condition (above) and an example choice after selecting “Times Chosen in Round 1” if three other players had answered the question (below).

Participants then proceeded through the 100 binary choice questions based on four different general-knowledge style categories. Participants had fifteen seconds to answer each question. On each question, participants could pick one of two answers (one correct and one incorrect), or choose to “Ask Someone Else,” in their group (Fig. 1). Choosing one of the two answers disabled the “Ask Someone Else” button for that question, and the participant received 1 point if their chosen answer was the correct one, and zero points if their chosen answer was incorrect. Alternatively, choosing “Ask Someone Else” allowed participants to see information about the other participants (henceforth ‘demonstrators’) in their group who did answer that question, i.e. who picked one of the two alternative answers, and copy one of those demonstrators’ answers. The specific demonstrator information participants saw after choosing to “Ask Someone Else” depended on which condition they were assigned to, detailed below. Participants could then choose a demonstrator whose answer they could use for that question (Fig. 2). If the chosen demonstrator answered the question correctly, the copying participant also scored a point for that question. If the demonstrator was incorrect, the copying participant did not score a point for that question. In the eventuality that everyone in a group chose to copy on a single question, participants would be shown a message telling them that because everyone chose to copy on that question, no one could score a point (as there were no answers to copy), however this never happened during data collection. No one received feedback on whether their answers to specific questions were right or wrong at any point in the game, only their final score at the end of the quiz. Participants were told explicitly that they could ‘Ask Someone Else’ on any question, and in doing so they could score a point for the question by choosing someone who had answered that question correctly. Participants were explicitly told that points obtained via copying would contribute to their final score, and if they scored over 85% they would receive a $20 bonus payment. No deception or manipulation of cues was employed at any time.

### Experimental conditions

The experimental conditions are summarised in Table 1. Each group of ten participants was assigned to one of the three conditions (Control, Prestige or Success—labelled A, B and C respectively in the raw data), with ten groups in each condition, giving a between-group design. Within each condition, there were two rounds of quiz questions. Round 1, comprising the first 40 questions, provided participants with a single cue upon which to choose a demonstrator from whom to copy, if they chose to “Ask Someone Else”. Round 2, comprising the subsequent 60 questions, gave participants a choice of two types of cues from which to then select demonstrators, as explained below.

**Table 1:** Information available to participants when choosing to copy in each round and condition.

	Control	Prestige	Success
Round 1 information provided when choosing to copy (first 40 Questions):	Player IDs of the demonstrators **(irrelevant cue)**	Scores of the demonstrators so far **(success cue)**	Scores of the demonstrators so far **(success cue)**
Round 2 choice of information when choosing to copy (final 60 Questions):	Either the player IDs of the demonstrators **(irrelevant cue)** OR the number of times the demonstrators were copied in Round 1 of this condition **(unreliable prestige cue)**	Either the player IDs of the demonstrators **(irrelevant cue)** OR the number of times the demonstrators were copied in Round 1 of this condition **(reliable prestige cue)**	Either the scores of the demonstrators from Round 1 of this condition **(success cue)** OR the number of times the demonstrators were copied in Round 1 of this condition **(reliable prestige cue)**

In the Control Condition, Round 1 information consisted of the random Player IDs of the demonstrators. This was an irrelevant cue, as it provided no information regarding the ability, knowledge or success of the demonstrators. In Round 2, participants could choose to use either the Player ID (again, an irrelevant cue), or the number of times that each demonstrator was copied in Round 1 of that condition. The latter is a prestige cue (frequency of being copied), but is unreliable because it is based on choices from Round 1 that were based on the irrelevant Player ID cue. Here we predict that participants should exhibit no preference between the irrelevant cue, and the unreliable prestige cue that is based on an irrelevant cue, as they are both irrelevant to quiz success.

In the Prestige Condition, Round 1 information consisted of the scores of the demonstrators up to that point in the quiz. This is a relevant success cue, as it indicates whether that participant is likely to get the current question correct. In Round 2, participants could choose to use either the Player ID of the demonstrators (irrelevant cue) or the number of times the demonstrators were copied in Round 1 of that condition. This is a reliable prestige cue, as it is based on choices from Round 1 which were informed by demonstrator success. Here we predict that participants should select the reliable prestige cue over the irrelevant cue, as the former provides some indication of the success of the demonstrator.

In the Success Condition, Round 1 information consisted of the scores of the demonstrators up to that point in the quiz, as in the Prestige condition. This again is a relevant success cue. In Round 2, however, participants could choose to use either the scores of the demonstrators from Round 1 (a success cue), or the number of times the demonstrators were copied in Round 1. This is again a reliable prestige cue, as it is based on choices from Round 1 that are informed by demonstrator success. However, here we predict that participants should select the direct success cue (score) rather than the indirect prestige cue (number of times copied), as the former should be more reliable than the latter.

### Analysis and preregistered predictions

All analyses were carried out using Bayesian multi-level models with the Rethinking package version 1.90^19^ in R version 3.6.0^20^. Our preregistration document is available at (https://osf.io/3tnv4), and our analysis scripts and data are available at (www.github.com/lottybrand/Dallinger_Analysis). Each model corresponding to each prediction is also included in the supplementary material. Model parameters are interpreted as providing evidence of an effect on the outcome if their 89% credible interval (CI) did not cross zero, with 89% CIs the default value in the Rethinking package. Priors were chosen to be weakly regularising, in order to control for both under and overfitting the model to the data. Convergence criteria such as effective sample sizes and Rhat values were used to check for appropriate model convergence throughout, and trace plots were inspected for signs of incomplete mixing when necessary.

### Predictions

Our two hypotheses (see above) generate the following specific preregistered predictions:

1. When choosing to “Ask Someone Else” in Round 1 of the Prestige and Success Conditions, participants preferentially copy the highest-scoring demonstrator. This is an assumption-check to make sure that subsequent copying frequency cues are genuine signals of performance.

To test prediction 1, we ran a multi-level binomial logistic regression. The binary outcome variable was whether participants chose the highest scoring participant or not, when they were making a copying decision (and when this information was available to them, n = 1516). We included varying intercepts for participant and group. Copying decisions were only recorded as copying the most successful if there was more than one model to choose from, and if there was variation in the models’ scores (i.e. instances where all available scores were tied were not counted).

2. When choosing to “Ask Someone Else” and copy frequency is chosen in Round 2 of the Prestige and Success Conditions, participants choose to copy the most-copied demonstrator. This is an assumption-check to make sure that people are actually employing prestige bias when it is potentially useful. No directional prediction is made about Round 2 of the Control Condition because there prestige cues are unreliable.

To test prediction 2, we ran a multi-level binomial logistic regression. The binary outcome variable was whether participants chose the most-copied participant available to them or not, when they were making a copying decision (and when this information was available to them, n = 982). We included varying intercepts for participant and group. Copying decisions were only recorded as copying the most prestigious if there was more than one model to choose from, and if there was variation in the models’ prestige scores (i.e. instances where all available models had been copied an equal number of times were not counted).

3. In Round 2 of the Control Condition, of those participants who choose to “Ask Someone Else”, there is no preference between choosing to view demonstrators’ player ID (irrelevant cue) or copy frequency (unreliable prestige cue).
4. In Round 2 of the Prestige Condition, of those participants who choose to “Ask Someone Else”, there is a preference for choosing to view demonstrators’ copy frequency (reliable prestige cue) over player ID (irrelevant cue).
5. In Round 2 of the Success Condition, of those participants who choose to “Ask Someone Else”, there is a preference for choosing to view demonstrators’ score (success cue) over copying frequency (reliable prestige cue).

To test predictions 3, 4 and 5, we ran a multi-level binomial logistic regression. The binary outcome variable was whether participants chose to view prestige information or not, when they were choosing to copy in Round 2 in all conditions (n = 1701). We included varying intercepts for participant, group and condition.

6. The overall frequency of copying (i.e. choosing to “Ask Someone Else”) in Round 2 is higher in the Success Condition than the Prestige Condition, and higher in the Prestige Condition than in the Control Condition. This is because the Success Condition provides direct success information, the Prestige Condition provides copying cues that are only indirectly linked to success information, and the Control Condition provides no relevant direct or indirect success information.

To test prediction 6, we ran a multi-level binomial logistic regression. The binary outcome variable was whether participants chose to copy or not in Round 2 (n = 13,709). We included varying intercepts for participant, group and condition. We then computed contrasts between each of the conditions to check for differences in likelihood of choosing to copy on any given question.

7. Participants’ score is higher in Round 2 of the Success Condition than the Prestige Condition, and higher in the Prestige Condition than the Control Condition. This follows from prediction 5, assuming that better access to success information allows better success-biased social learning, and improved performance, compared to prestige-biased social learning, which in turn is better than copying decisions based on irrelevant cues.

To test prediction 7, we ran a multi-level linear regression. The outcome variable was each participant’s total score on the quiz (n = 225). We included varying intercepts for group and condition. We then computed contrasts between each of the conditions to check for differences in score between conditions. We also did the same for score in Round 2 only, with varying intercepts for group and condition.

### Ethics approval

All tasks were given ethical permission by the University of Exeter Biosciences ethics committee.

## Results

### Prediction 1 (assumption check)

As predicted, participants preferentially copied the highest-scoring demonstrator in Round 1 of the Prestige and Success Conditions. When participants chose to copy others, and chose to view success information, they overwhelmingly chose to copy the highest scoring participant available to them (mean coefficient estimate: 2.81, 89% CI: 2.42, 3.25, this corresponds to participants choosing the highest scoring model 94.3% of the time on the probability scale).

### Prediction 2 (assumption check)

As predicted, participants preferentially copied the most prestigious (i.e. most copied) demonstrator in Round 2 of the Prestige and Success Conditions. When participants chose to copy others, and chose to view prestige information, participants overwhelmingly chose to copy the most prestigious demonstrator available to them (mean coefficient estimate: 3.71, 89% CI: 3.03, 4.51, this corresponds to participants choosing the most copied model 97.6% of the time on the probability scale).

### Predictions 3, 4 and 5 (key hypothesis tests)

**Figure 3:**
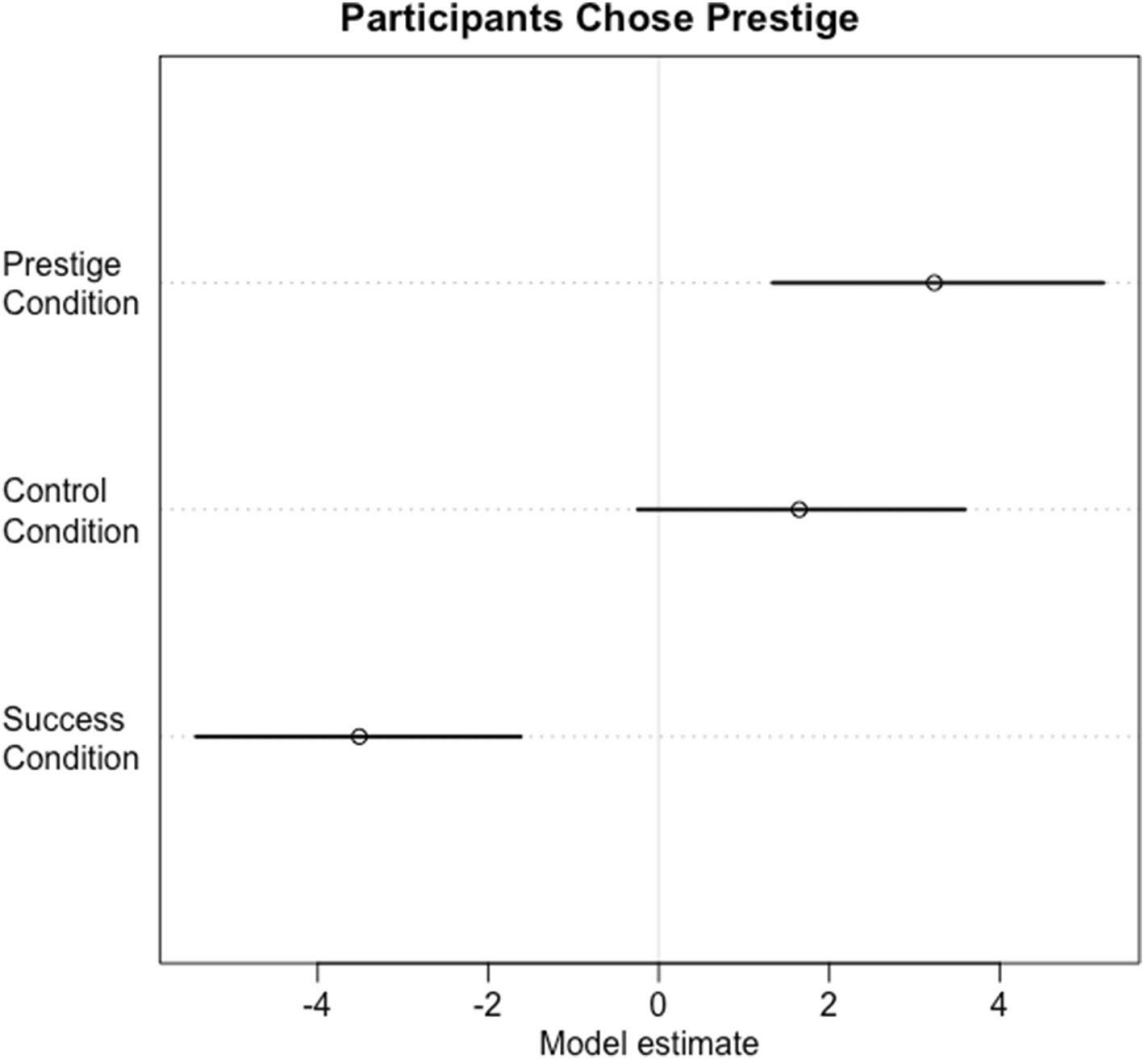
Model estimates for choosing prestige information (number of times demonstrators were copied in Round 1) compared to the alternative information in Round 2 of the Prestige, Control and Success Conditions. The alternative information for the Control and Prestige Conditions was irrelevant Player ID, and the alternative information for the Success Condition was success (score in the quiz). Participants therefore only reliably chose prestige when it was based on relevant information, and when success was not available.

As predicted, in Round 2 of the Control Condition, there is not strong evidence that participants showed a preference for (unreliable) prestige information over the irrelevant cues, assuming chance level is 0.5 probability of picking either option (Fig. 3; mean: 1.57, 89% CI: − 0.22, 3.45). Also as predicted, in Round 2 of the Prestige Condition participants clearly chose to use the reliable prestige cues over the irrelevant cues (Fig. 3; mean: 3.15, 89% CI: 1.32, 5.09). Finally, and also as predicted, prestige cues were not preferred over direct success cues in Round 2 of the Success Condition; instead direct success cues were preferred (Fig. 3; mean: − 3.62, 89% CI: − 5.47, − 1.81).

### Prediction 6

As predicted, participants chose to copy more in Round 2 of the Prestige Condition (13.5% copying rate, n = 4529) and Success Condition (15% copying rate, n = 4380) than in Round 2 of the Control Condition (9% copying rate, n = 4800). This was confirmed by computing contrasts between the estimates of the three conditions (mean difference between Control and Prestige: 1.04, 89% CI: 0.27, 1.79. Mean difference between Control and Success: 1.11, 89% CI: 0.35, 1.87. Mean difference between Success and Prestige: − 0.07, 89% CI: − 0.82, 0.67).

### Prediction 7

In contrast to our prediction, participants did not reliably score higher in the Prestige Condition (mean score 69.7%) and Success Condition (69.5%) than the Control Condition (66.9%). This was confirmed by computing contrasts between the estimates of the three conditions (mean difference between Control and Prestige: 0.51, 89% CI: − 0.53, 1.56; Mean difference between Control and Success: 0.47, 89% CI: − 0.56, 1.46; Mean difference between Success and Prestige: 0.04, 89% CI: − 1.00, 1.05). There was also no difference in score when analysing Round 2 separately.

### Exploratory analyses

Exploratory analyses were conducted to follow up on the lack of difference across conditions in score for Prediction 7. Interestingly, we found that individual copying rate did predict score for participants in the Prestige and Success conditions, suggesting that social learning was adaptive in these two conditions (mean coefficient estimate: 0.16, 89% CI: 0.10, 0.22). There was not strong evidence that copying rate predicted score in the Control Condition (mean coefficient estimate: 0.1, 89% CI: 0.00, 0.19). Furthermore, social learning seems to have been underused in our sample: of the 225 participants, most either never copied (n = 68) or copied on less than 10 questions (n = 155). Only 19 copied on over half of the questions, with one individual copying on 96 out of 100 questions.

**Figure 4:**
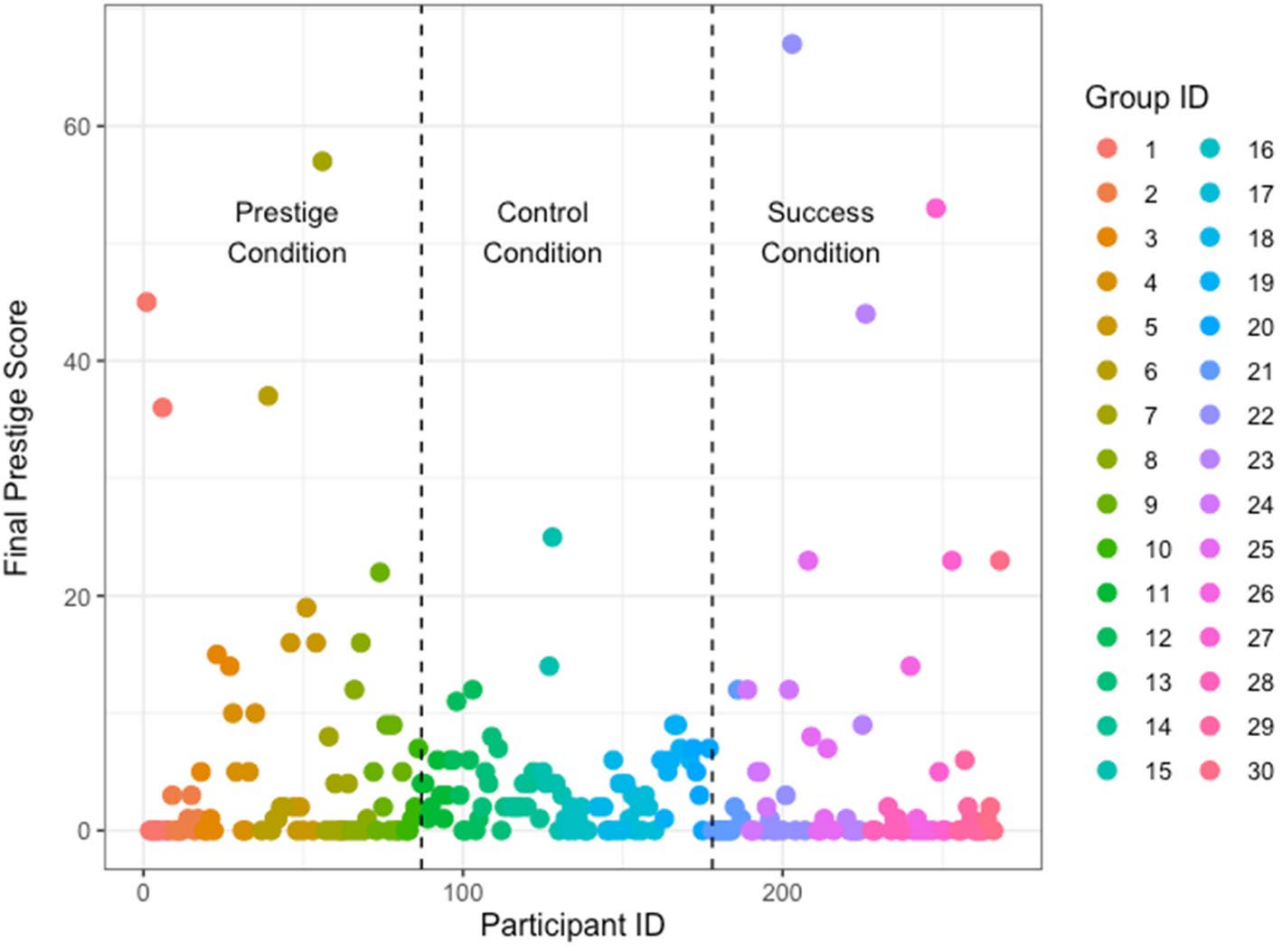
The total number of times each participant was copied (i.e. their ‘prestige’) across all conditions, clustered by group as indicated by different colours. The dashed lines demarcate the experimental conditions, Prestige, Control and Success from left to right. Groups 1–10 were in the Prestige Condition, 11–20 were in the Control, and 21–30 were in the Success Condition. The majority of participants were never copied, or were copied under 10 times. A minority of participants were copied 20–40 times, and just 5 participants were copied over 40 times.

**Figure 5:**
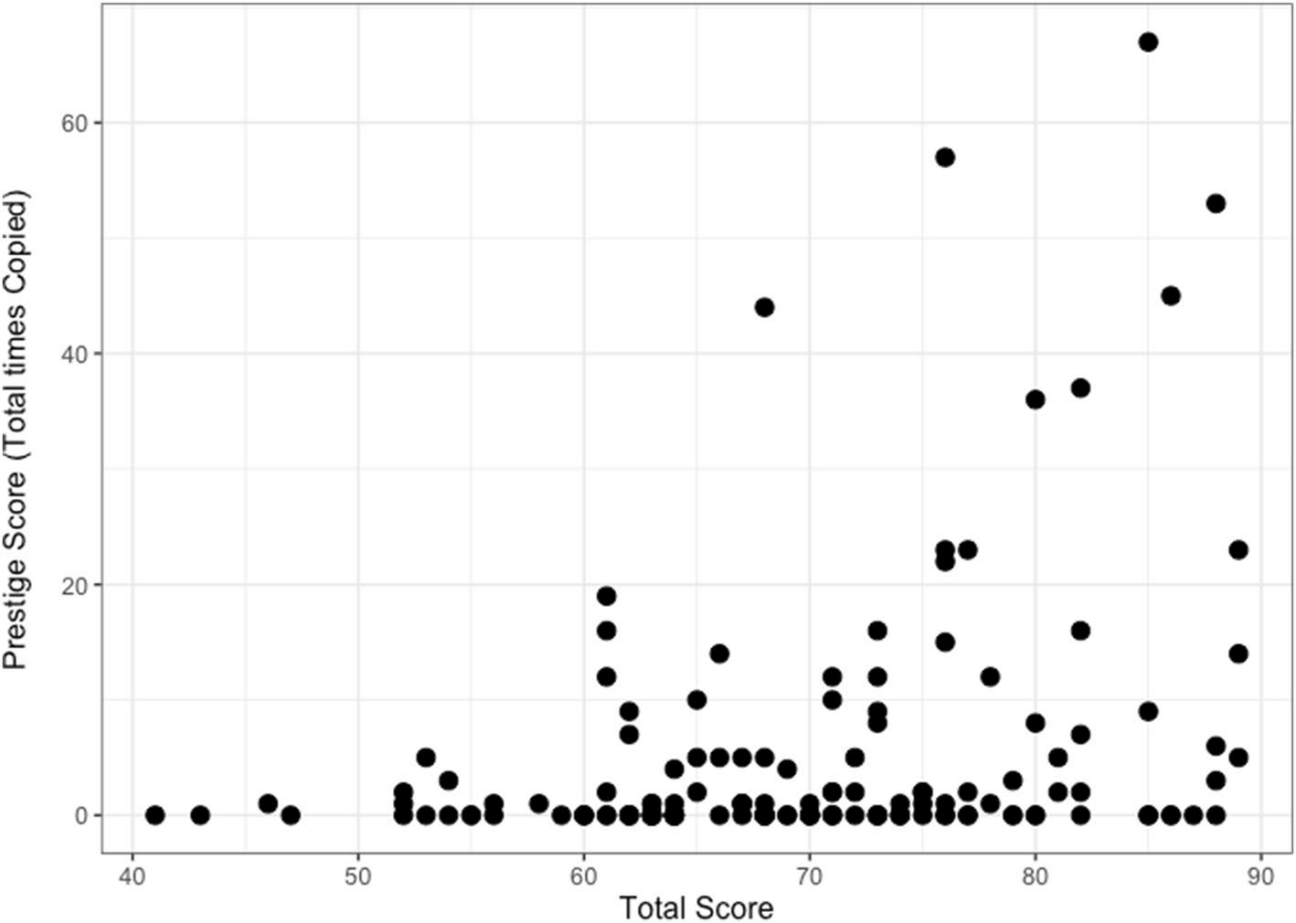
Relationship between participants’ total score and their ‘prestige score’ (i.e. total number of times copied) in the Prestige and Success Conditions, where score information was available either directly or indirectly.

Further exploratory analyses examined the emergence of prestigious individuals. The majority of participants were either never copied (n = 91), or copied less than 10 times (n = 106). A minority were copied between 20 and 40 times (n = 7) and only 5 participants were copied over 40 times. This is shown in Fig. 4, which displays prestige score clustered by quiz group. In 16 out of 20 groups in the Prestige and Success conditions in which success information was available, a ‘prestigious’ individual emerged, in that the most copied person was copied more than ten times. In the Control condition where success information was unavailable, a prestigious individual emerged in only 2 of 10 groups, and the top prestige score was tied in half of the groups (5/10), compared to only 3/20 times in the other conditions. As expected, prestige score was related to success in the quiz, in that total quiz score predicted prestige score, in the conditions in which success information was available (mean estimate: 0.26, 89% CI: 0.14, 0.38, see Fig. 5).

## Discussion

In this study we sought to experimentally test two key but previously untested assumptions underlying the theory of prestige-biased social learning^1^: that prestige information is only used when it provides a reliable signal of success, and that prestige information is only used when direct success information is unavailable. We had groups of participants complete an online quiz with the option to copy other people’s answers based on different cues. Our prestige cues were the number of times that participants had been copied earlier in the quiz. Importantly, prestige cues were an emergent property of participants’ behaviour during the experiment; no deception or manipulation of prestige cues was employed at any time. In support of both of our preregistered hypotheses, we found evidence that prestige bias was used when previous copiers had access to reliable success information such that the prestige cues provided a cue of success, and when direct success information was unavailable. This lends weight to arguments that humans use sophisticated and adaptive social learning strategies to acquire useful information even when demonstrator characteristics (e.g. success) are not immediately available, and that people are sensitive to the relative costs and benefits of alternative social learning strategies (e.g. success bias vs prestige bias).

In line with predictions one and two (our assumption checks), when participants chose to copy, they reliably chose to copy the highest scoring demonstrator out of those available, and when prestige information was available, they reliably chose to copy the most copied demonstrator out of those available. This is consistent with previous experimental studies of social learning showing that humans adaptively use success bias in a variety of tasks^21,22^, although we believe it is the first to demonstrate that the most prestigious participants are copied when success information is unavailable. Participants also chose to copy more often in the Prestige and Success Conditions compared to the Control Condition in which only irrelevant characteristics were available to copiers. This is in line with cultural evolution theory and previous studies showing that social learning is employed adaptively^23, 24^.

Interestingly, against our prediction, participants did not score higher in the Prestige and Success Conditions compared to the Control Condition. However, exploratory analyses showed that social learning rates did predict score in the Prestige and Success Condition, but not the Control Condition. We also found large individual variation in social learning rates, in that most people never copied, or copied under 10 times, but a minority of people copied on over half the questions. This supports previous findings of individual variation in social learning strategies^25^ and that pay-off biased social learning is adaptive but underused^22,26,27^. Curiously, the top three scorers, who each scored over 90%, were all in the Control Condition. Two of these high scorers were pure asocial learners as they never copied an answer from another participant. This peculiarity—that the three most knowledgeable players were in the control condition—demonstrates that success information has to be available for prestige bias to evolve. Although these players were extremely successful, they did not become prestigious and were only copied 2, 3 and 5 times respectively.

The current study reported here was preceded by two pilot studies (see Supplementary Information for full details of the pilots). The first of these pilot studies used the same knowledge-based quiz and the same condition structure as the current study reported here. However, in contrast to the current study, the irrelevant characteristic in the control condition of the first pilot consisted of the demonstrators’ favourite hobbies, rather than the randomly generated Player IDs that we used here. In contrast to the current study, and against our predictions, participants in the control condition of the first pilot chose to copy based on the irrelevant cue just as much as they chose to copy based on prestige. Although we assumed that hobbies would be unrelated to quiz performance and thus constitute an irrelevant cue, some hobbies (e.g. gardening) were actually associated with higher quiz knowledge, and others with lower knowledge. Although participants were unaware of this correlation, hobbies may have been inadvertently perceived as reliable cues by the participants, or simply perceived as more appealing information to view. It is also possible that this unexpected finding represents a form of “normative prestige”, where participants wanted to copy players who had similar hobbies or viewpoints to them^28^. However, the copying rates in the first pilot study were unexpectedly low, and so this result should be interpreted with caution. Written feedback suggested that participants were reluctant to ‘copy’ answers in the quiz because the term ‘copy’ has negative connotations such as cheating on tests. We therefore used the less culturally loaded phrase “Ask Someone Else” for the current study. More information on the previous study can be found in the supplementary material.

The second pilot study involved an abstract social learning task that we ran with participants in Finland. Participants interacted in live online groups to guess a randomly generated hidden pattern by revealing squares in the grid. As in the current study, they could choose to copy other participants’ guesses across similar conditions that manipulated cue information. Consistent with results of the current study, participants used prestige-biased copying least when success information was freely available. When success information was costly to acquire, participants used prestige-biased copying as much as they did when it was the only information available. This additional study illustrates that our findings are not culturally-specific, replicating in a non-English-language participant sample, and that they are not task specific, extending to a non-verbal pattern-matching task.

Finally, it is worth comparing these online results to a complementary study with already-established volunteer groups in Cornwall^29^. In our Cornwall based study we found that participants nominated the most successful (i.e. highest quiz score), rather than the most prestigious (i.e. highly respected and influential to the group), person to complete a bonus round of the quiz on behalf of the group. Importantly, in contrast to the current study, prestige had been developed in those groups based on the previous interactions of their group, e.g. their chess club or rowing club, and not on who had been copied during the quiz. Therefore, prestige was unrelated to the quiz. This result suggests that people may be sensitive to the source of a demonstrator’s prestige, and that prestige-bias may be domain-specific, although this needs to be tested experimentally. Furthermore, it demonstrates that prestige hierarchies can emerge over time for a variety of domains, providing a potential shortcut for social learning, at least within the domain in which prestige was acquired.

One distinction that is lacking in Henrich and Gil-White’s^1^ theory of prestige is that between knowledge and skill. Indeed, the authors repeatedly use the terms “knowledgeable” and “skillful” interchangeably. Nonetheless the two can be distinguished, with declarative, explicit knowledge concerning beliefs (e.g. statements about animal migratory patterns) and implicit, procedural skills concerning the ability to perform motor actions (e.g. wood-working)^30,31^. Given this distinction, our task concerned declarative knowledge, and so our results demonstrate that success and prestige bias can operate in this domain, at least when success information is explicitly available. While many social learning opportunities since the evolution of language would have relied on declarative knowledge exchange via language and teaching, many others involve procedural skills. Yet most social learning experiments to date use tasks that do not distinguish between, or explicitly compare, the copying of decisions, products, procedural knowledge or declarative knowledge. Future studies should test hypotheses related to prestige biased social learning, as well as other social learning strategies, in a range of tasks, including those related to procedural skills, and via a range of processes (e.g. language-mediated teaching).

Prestige biased social learning, in acting as an efficient short-cut to acquire adaptive information, can facilitate cumulative cultural evolution, often argued to be responsible for our species’ ecological success. However, it is important to note that in our task, success information was explicitly labelled as such, as well as being accurate and free to access. For prestige-biased social learning to be adaptive, at least some members of the population have to be able to identify a successful individual to begin with (the equivalent in our study of Round 1, where success information was available). Further research is needed to determine whether or not success-biased and prestige-biased social learning can support cumulative cultural evolution even when traits are so opaque as to make success or payoff information difficult to acquire without some level of causal understanding of the technology to begin with^32^. In these instances, other mechanisms such as language-mediated teaching may be necessary, in which the reasons for a demonstrator’s success can be communicated explicitly.

In summary, we find evidence that two key assumptions of prestige-biased social learning theory are supported in an online, knowledge-based task, in which participants could decide whether, when, and from whom to copy in real-time. Specifically, participants use prestige-biased social learning when success information is unavailable, and when the prestige cue is an indirect measure of success. Importantly, prestige cues were an emergent property of participants’ behaviour during the experiment and were not artificially produced by us for the purpose of the experiment. However much more work needs to be done to a) understand the domain specificity and generalizability of prestige cues and to b) demonstrate that prestige-bias operates in domains in which success cues are opaque or obscured.

## Supplementary information

Supplementary file1 (PDF 3157 kb)

## Data Availability

www.github.com/lottybrand/DallingerAnalysis.
