# Supplementary material for: The emergence and adaptive use of prestige in an online social learning task
Source: Sci Rep. 2020 Jul 21;10:12095. doi: 10.1038/s41598-020-68982-4 (PMC7374563; doi:10.1038/s41598-020-68982-4)
Supplement: Supplementary file 1 — Supplementary file1 (PDF 3157 kb) [file 41598_2020_68982_MOESM1_ESM.pdf]

Supplementary material

The emergence and adaptive use of prestige in an online social learning task

C O Brand[1*]
S Heap[2]
TJH Morgan[3,4]
A Mesoudi[1]

[1] Human Behaviour and Cultural Evolution Group, Department of Biosciences, College of Life & Environmental Sciences, University of Exeter's Cornwall Campus. [*] Corresponding author: c.brand@exeter.ac.uk
[2] Department of Biological and Environmental Science, University of Jyväskylä
[3] School of Human Evolution and Social Change, Arizona State University,
[4] Institute of Human Origins, Arizona State University

The following supplementary material contains the code for each model in the analysis, and information on two pilot studies that informed the design of the final study reported in the main manuscript.

Contents:

pp 2 – 3: Model code from main study
pp 4 – 10: Pilot Study 1
pp 11 - 16 : Pilot Study 2
pp 17 – 18 : Consent & Information sheet from main study
pp 19 – 39: Full Quiz Questions from main study

Here we include each model used for each prediction in the paper. Full code and details are available at www.github.com/lottybrand/Dallinger_Analysis

Model 1. Testing prediction 1: "participants copied the most successful model when available"

```
model1 <- map2stan(
  alist(
    copied_successful ~ dbinom(1, p),
    logit(p) <- a + a_p[pptIndex]*sigma_p + a_g[groupIndex]*sigma_g,
    a ~ dnorm(0,2),
    a_p[pptIndex] ~ dnorm(0,1),
    a_g[groupIndex] ~ dnorm(0,1),
    sigma_p ~ dcauchy(0,1),
    sigma_g ~ dcauchy(0,1)
  ),
  data=scoreChoice, constraints=list(sigma_p="lower=0", sigma_g="lower=0"),
  warmup=1000, iter=4000, chains=3, cores=3)
```

Model 2. Testing prediction 2: "participants copied the most prestigious model when available"

```
model2 <- map2stan(
  alist(
    copied_prestigious ~ dbinom(1, p),
    logit(p) <- a + a_p[pptIndex]*sigma_p + a_g[groupIndex]*sigma_g,
    a ~ dnorm(0,2),
    a_p[pptIndex] ~ dnorm(0,1),
    a_g[groupIndex] ~ dnorm(0,1),
    sigma_p ~ dcauchy(0,1),
    sigma_g ~ dcauchy(0,1)
  ),
  data=prestigeChoice, constraints=list(sigma_p="lower=0", sigma_g="lower=0"),
  warmup=1000, iter=4000, chains=3, cores=3 )
```

Model 3. Testing predictions 3, 4, 5: "participants in the prestige condition were more likely to choose to view prestige information than the alternative, participants in the success condition were more likely to choose success information than prestige."

```
model3.1 <- ulam(
  alist(
    chosePrestige ~ dbinom( 1 , p ) ,
    logit(p) <- a[pptIndex] + g[groupIndex] + b[condsIndex] ,
    b[condsIndex] ~ dnorm( 0 , sigma_b ),
    a[pptIndex] ~ dnorm( a_bar , sigma_a ),
    g[groupIndex] ~ dnorm( 0 , sigma_g ),
    a_bar ~ dnorm( 0 , 1.5 ),
    sigma_a ~ dexp(1),
    sigma_g ~ dexp(1),
    sigma_b ~ dexp(1)
  ) , data=infoChosen_list, constraints=list(sigma_a="lower=0", sigma_g="lower=0", sigma_b="lower=0"), control=list(
adapt_delta=0.99, max_treedepth=13),
  warmup=1000, iter=9000, chains=3 , cores=3 , log_lik=TRUE )
```

Model 4. Testing prediction 6: participants copied most in the success condition, and more in the prestige than the control condition.

```
model4.4 <- ulam(
  alist(
    copied ~ dbinom( 1 , p ) ,
    logit(p) <- a_bar + a[pptIndex]*sigma_a + g[groupIndex]*sigma_g + b[condsIndex],
    b[condsIndex] ~ dnorm( 0 , 1 ),
    a[pptIndex] ~ dnorm( 0 , 1 ),
    g[groupIndex] ~ dnorm( 0 , 1 ),
    a_bar ~ dnorm ( 0, 1 ),
    sigma_a ~ dexp(1),
    sigma_g ~ dexp(1)
  ) , data=asocialOnly_list_2 , constraints=list(sigma_a="lower=0", sigma_g="lower=0"), control=list( adapt_delta=0.99, max_treedepth=13),
  warmup=1000, iter=5000, chains=3 , cores=3 , log_lik=TRUE )
```

Model 5. Testing prediction 7: participant score highest in the success condition, and higher in the prestige than the control condition.

```
model5 <- map2stan(
  alist(
    t_score ~ dnorm(mu, sigma),
    mu <- a + b[condsIndex] + g[groupIndex],
    a ~ dnorm(50,10),
    b[condsIndex] ~ dnorm(0,0.5),
    g[groupIndex] ~ dnorm(0,0.5),
    sigma ~ dexp(1)
  ), data = finalScore_list, chains=3)
```

Pilot Study 1: Qualtrics Quiz

This study was designed to be an initial test of our quiz, the copying rate of participants, and whether they would choose to view prestige information when copying over “irrelevant information” such as participants’ favourite hobbies.

METHODS:
General: Participants had to answer 100 binary choice quiz questions from four topics, World Geography, Language identification, Weight estimation and Art History (see below). Participants had the option of “copy someone else” if they did not know the answer.

Geography:

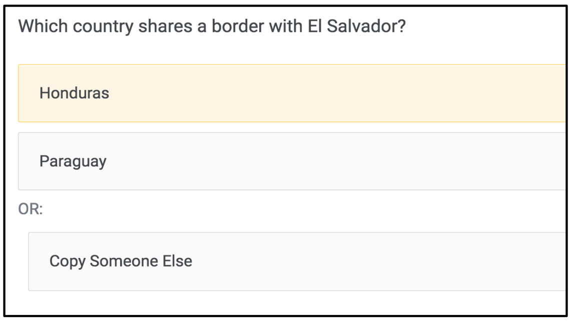
Which country shares a border with El Salvador?

Honduras

Paraguay

OR:

Copy Someone Else

Language identification:

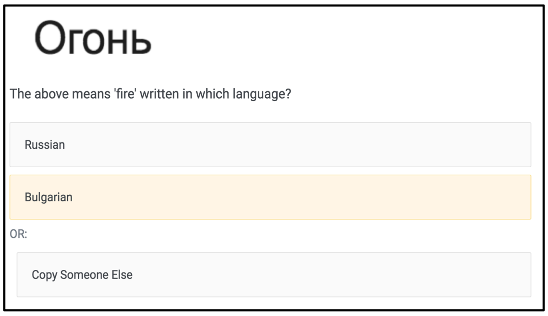
Огонь

The above means 'fire' written in which language?

Russian

Bulgarian

OR:

Copy Someone Else

Weight estimation:

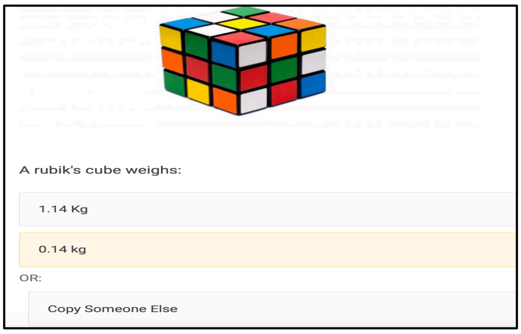
A rubik's cube weighs:

1.14 Kg

0.14 kg

OR:

Copy Someone Else

Art history

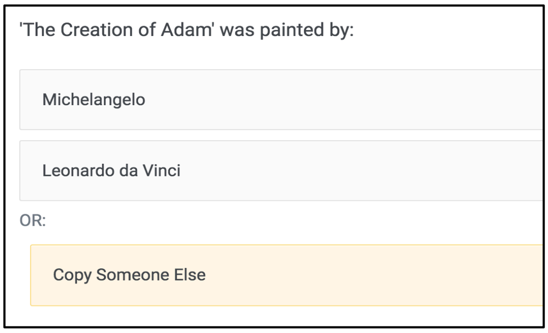
'The Creation of Adam' was painted by:

Michelangelo

Leonardo da Vinci

OR:

Copy Someone Else

Round 1: 100 participants answered the 100 quiz questions, with no option for social learning. At the end of the quiz, participants were asked questions about their favourite hobbies, favourite film, favourite music etc (see below).

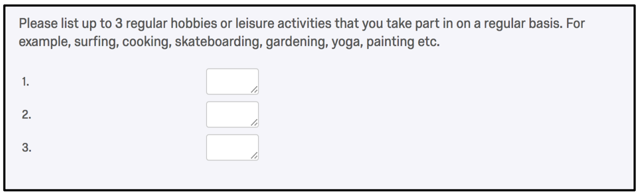
Please list up to 3 regular hobbies or leisure activities that you take part in on a regular basis. For example, surfing, cooking, skateboarding, gardening, yoga, painting etc.

1.

2.

3.

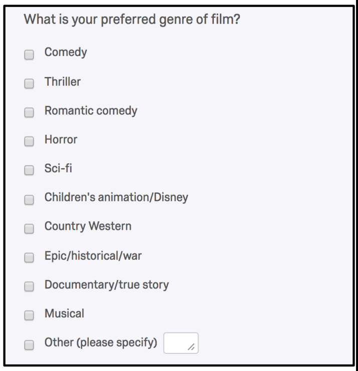
What is your preferred genre of film?

- Comedy
- Thriller
- Romantic comedy
- Horror
- Sci-fi
- Children's animation/Disney
- Country Western
- Epic/historical/war
- Documentary/true story
- Musical
- Other (please specify)

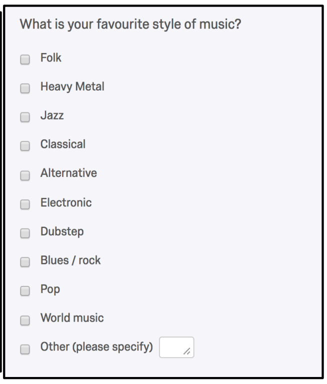
What is your favourite style of music?

- Folk
- Heavy Metal
- Jazz
- Classical
- Alternative
- Electronic
- Dubstep
- Blues / rock
- Pop
- World music
- Other (please specify)

Round 2: 100 participants split into two conditions. Condition A: participants can copy others’ answers based on music or hobbies. Condition B: participants can copy others’ answers based on hobbies or score. (See below).

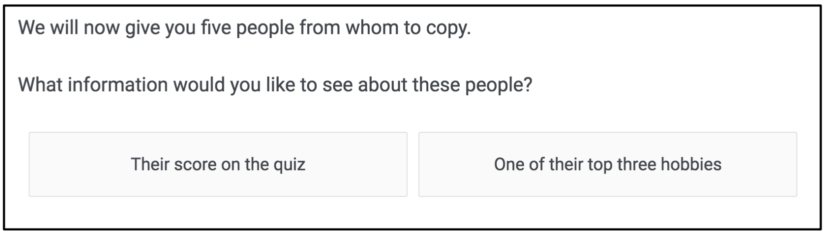

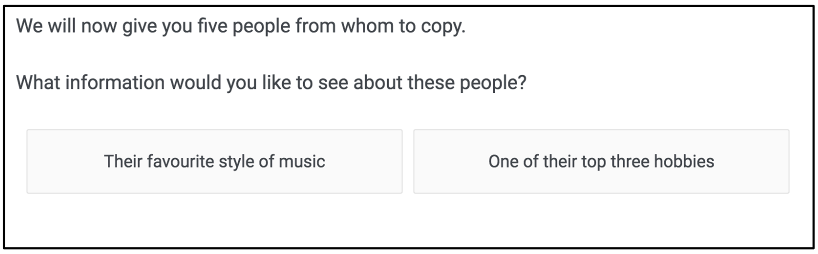

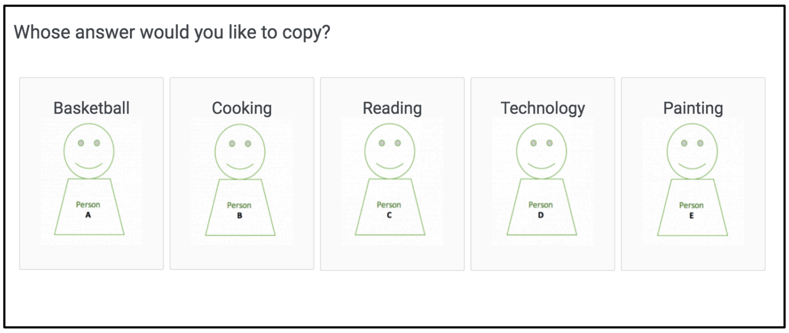

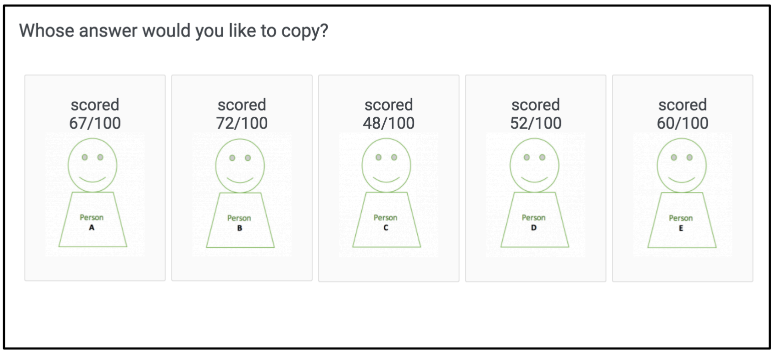

Round 3: 150 ppts, split into three conditions. Conditions A: can copy based on hobbies or number of times copied (in the previous round). Condition B: can copy based on hobbies or number of times copied. Condition C: can copy based on score or number of times copied:

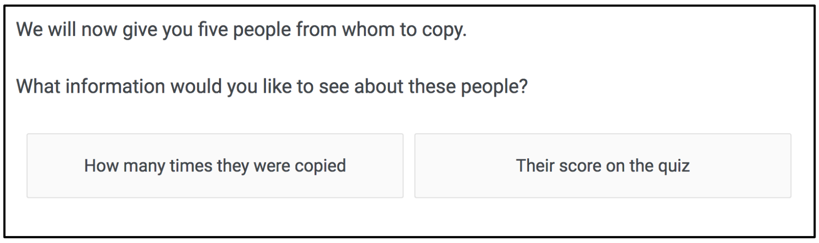

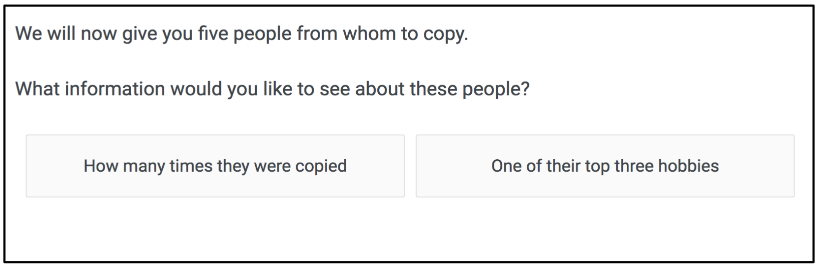

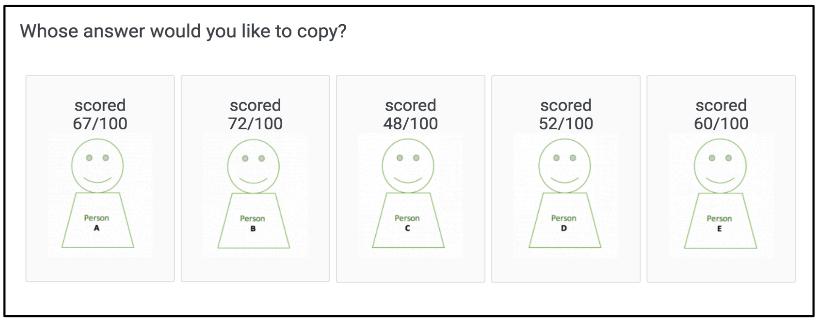

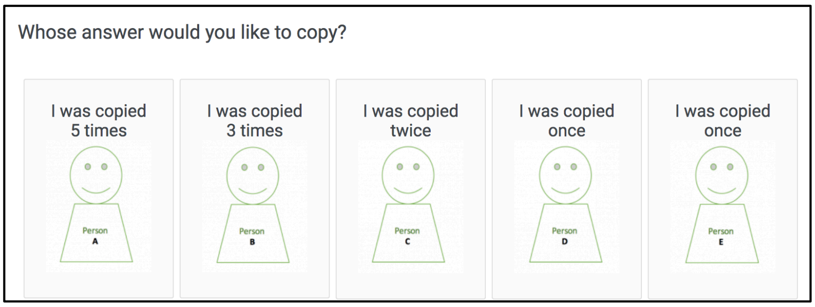

Predictions (preregistered at https://osf.io/g3mhj)

1. When participants choose to copy based on score, they will choose the highest scoring participant available to copy from
2. When participants choose to copy based on copy-frequency, they will choose to copy the most-copied participants
3. Participants choose to view the copy-frequency information more in Condition B than the other two conditions because i) in Condition B copiers can access success information, unlike Condition A where copiers only have access to irrelevant information, and ii) in Condition B copy frequency is the only relevant cue available, unlike condition C where direct pay-off information is also available
4. Copying rate will be higher in conditions B & C compared to A because copying can be based on success in Conditions B & C.
5. Participants perform better in Conditions B & C compared to Condition A because copying can be based on success information in Conditions B & C.

Analysis:

All data were analysed with multi-level models using Bayesian HMC estimation in the *Rethinking* package in R (McElreath 2016) and using a model comparison approach. Condition A was modelled as the baseline, thus parameter estimates for Conditions B and C are in comparison to Condition A. All parameter estimates are displayed with the default 89% confidence intervals .

Results:

Was prestige info used more in condition B compared to A & C?

Parameter estimates for likelihood of choosing Prestige information

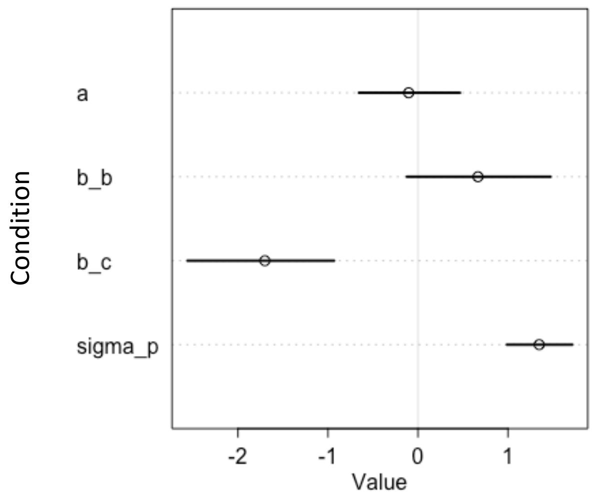

Proportion of times they chose prestige/score/hobby information

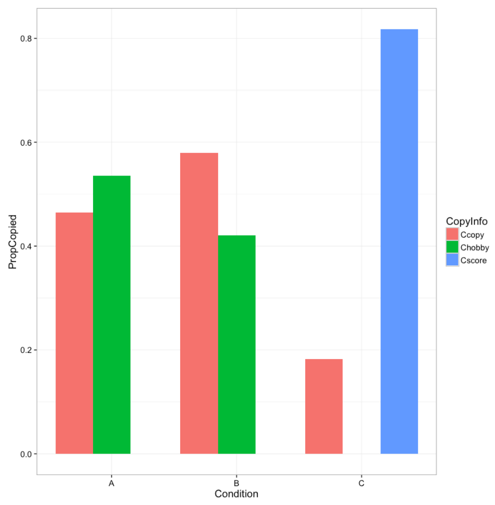

Likelihood of choosing prestige

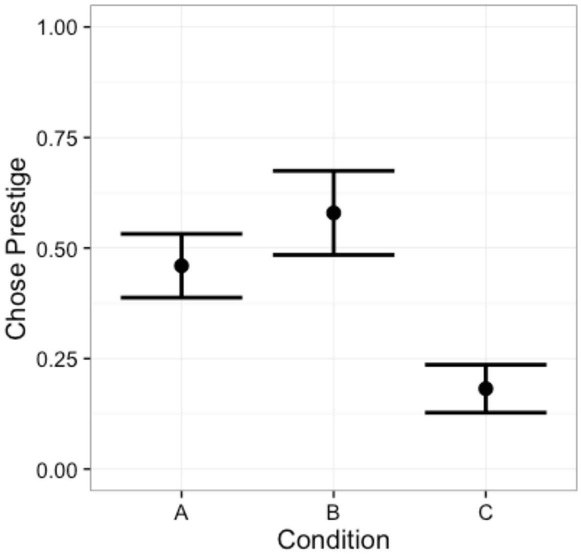

Did people copy more in Condition B?

Parameter estimates for copy frequency

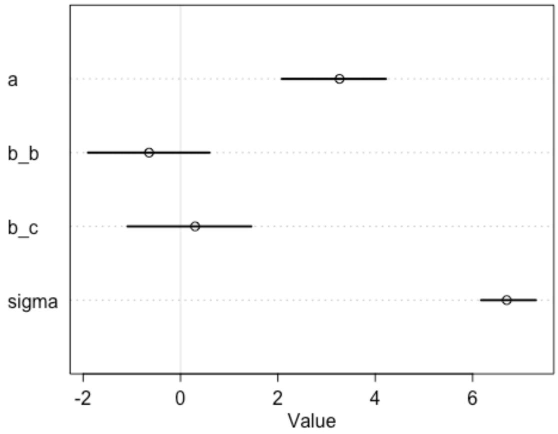

Proportion of times they chose to copy

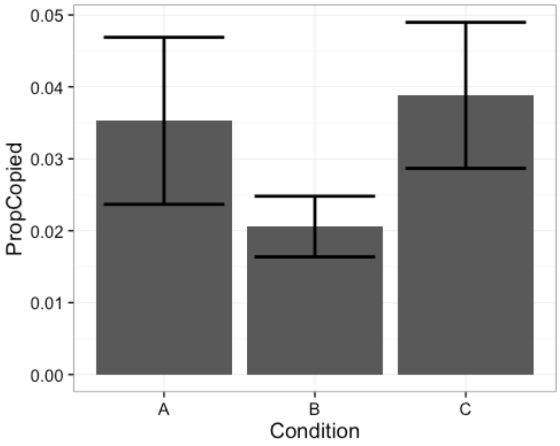

Did people score higher in condition B & C compared to A?

Parameter estimates for score

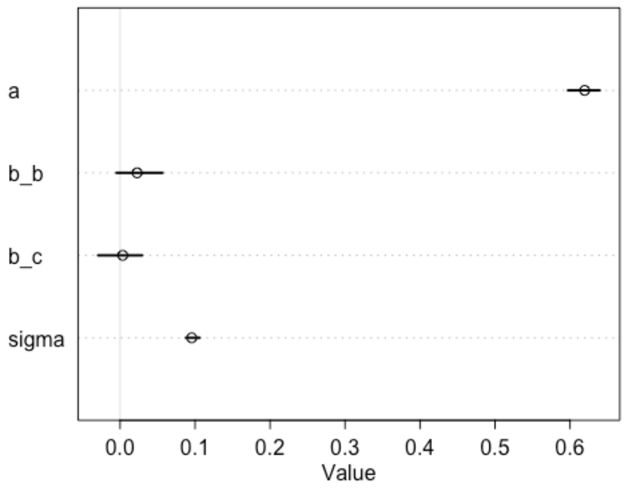

Participants' scores in each condition

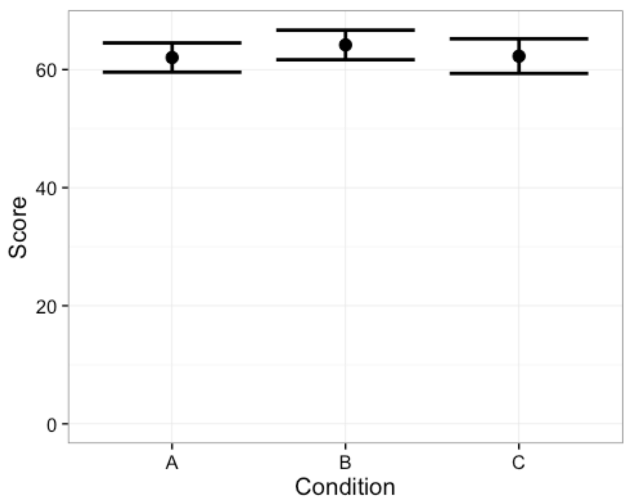

Did participants "copy the most copied"?

Parameter estimates for choosing the most copied

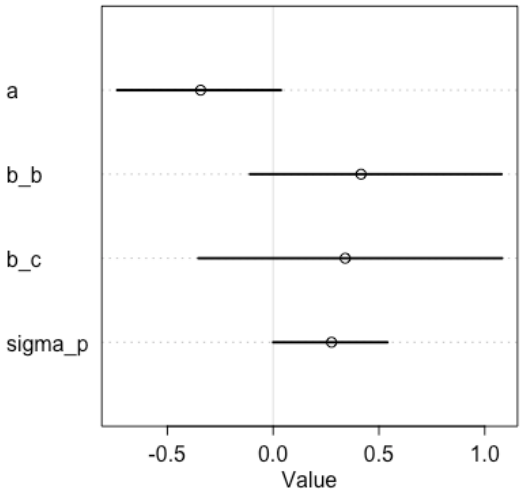

Did they copy the least copied?

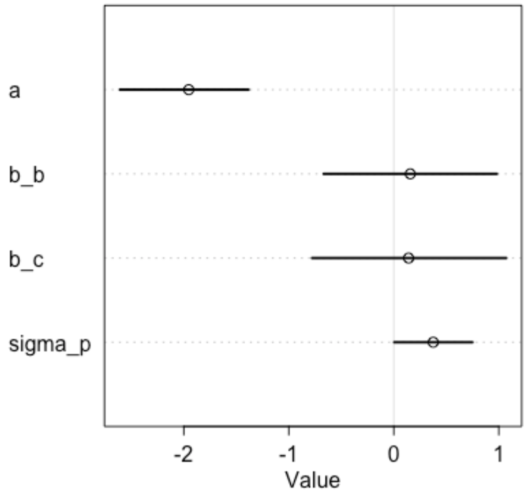

Did they copy the highest scorer?

Parameter estimates for choosing the most copied (only available in Condition C)

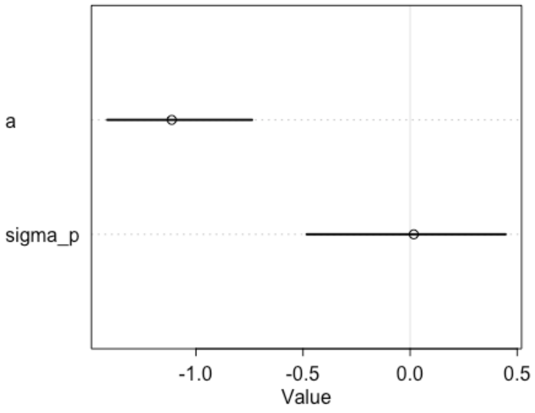

Did they copy the least copied?

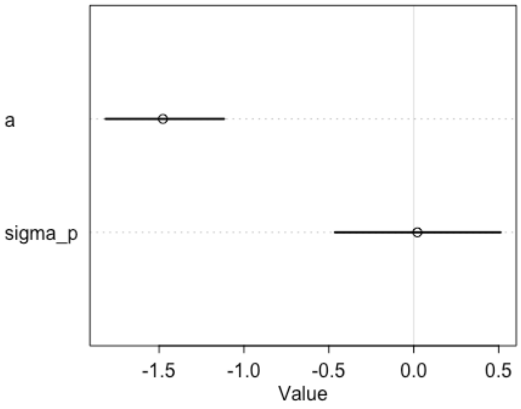

Pilot 1 Conclusions

- People choose to copy based on prestige less when pay-off information is freely available
- No difference between copying based on prestige or copying based on an "uninformative" trait like favourite hobby
- People copied very rarely (reported wanting to see how they did on their own, and copying is "bad")
- When they did use prestige information, they didn't "copy the most copied"… but they didn't copy at random (avoided the least copied). Equally they didn't choose to copy the highest scorer, but they avoided the lowest scorer.
- Participants weren't using the social information to their advantage (didn't gain a higher score when score information was freely available)
- Probably not incentivised enough to use copying to score highly on this task….

Pilot Study 2: Abstract task. (Designed by Dr. Stephen Heap)

This study was designed to test if participants would choose to view prestige information when success information was unavailable or costly, on an abstract task with no distracting "irrelevant characteristics" such as hobbies.

Methods:

1) The game involves guessing a **hidden pattern** in a grid, like this:

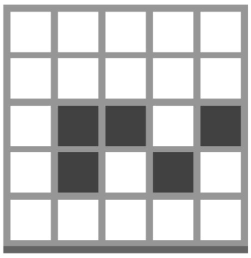

- The hidden pattern will be **randomly generated** by a computer
- The hidden pattern will **consist of 5 filled cells**
- Each filled cell will be in contact with at least one other filled cell (i.e. no isolated cells), and it will not be purposefully made to represent any picture or symbol

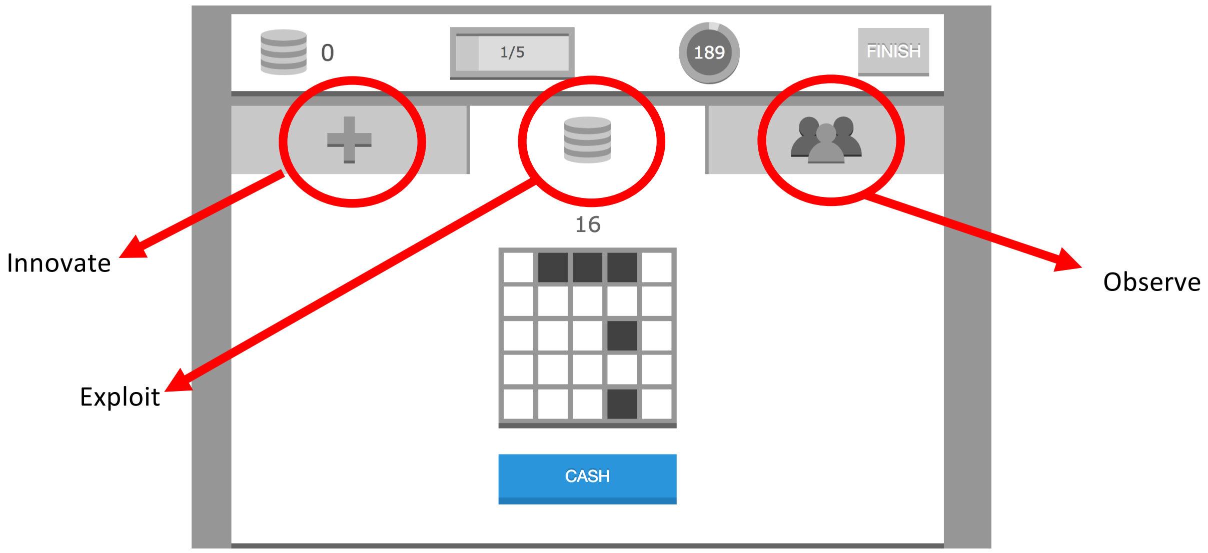

Condition A: Observe (100 ppts, 20 groups, 25 rounds):

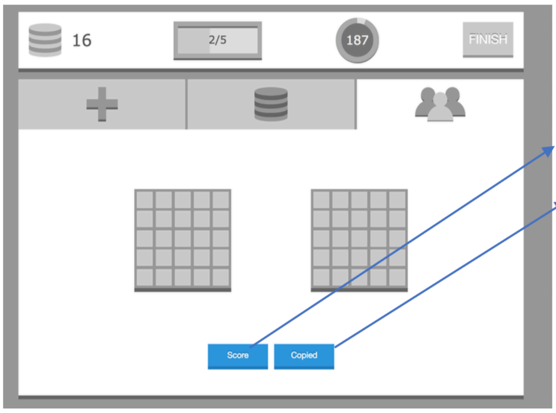

If you choose to copy someone else's pattern, you can either view their score, or the number of times they have been copied. Once you select to view either their score, or the number of times they have been copied, you have to select one of the patterns to copy.
This pattern is then added to your collection. You cannot cash that pattern in the same round as you copy it.

Condition B: Observe (100 ppts, 20 groups, 25 rounds):

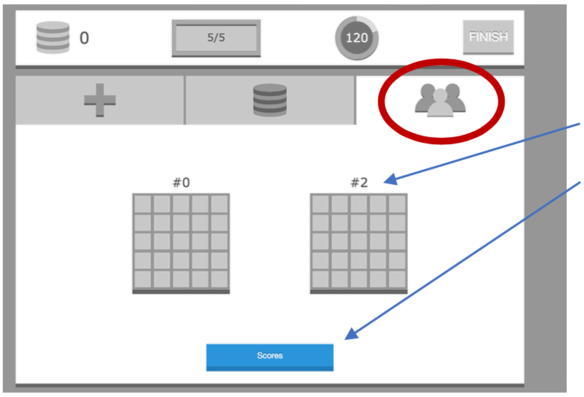

If you choose to copy someone else's pattern, you will be able to see grids with the number of times they have been copied. Alternatively you can select "scores" to view the scores of the grids instead.
If you select "scores" you have to then choose a grid to copy.
This pattern is then added to your collection. You cannot cash that pattern in the same round as you copy it.

Condition C: Observe (100 ppts, 20 groups, 25 rounds):

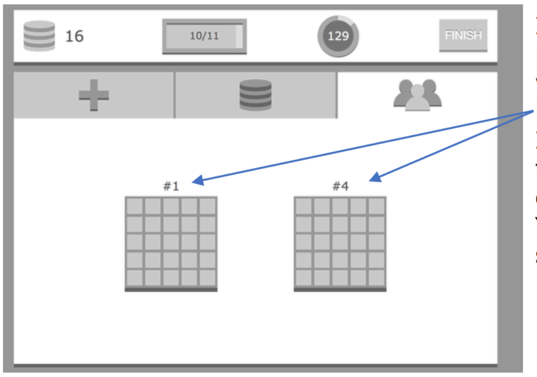

If you choose to copy someone else's pattern, you will be able to see grids with the number of times they have been copied displayed above.
If you select one of these grids, this pattern is then added to your collection.
You cannot cash that pattern in the same round as you copy it.

Predictions: (see pre-registration : [https://osf.io/5xh8k)](https://osf.io/5xh8k)

1. Ppts will choose score info more than prestige info in Condition A
2. Ppts will choose to copy the highest scoring pattern out of those available
3. Ppts will choose prestige information most in Condition C (as it is the only info available) and least in Condition A (as score info is freely available)
4. Ppts will choose to copy the pattern with the highest copy frequency out of those available
5. Ppts will score highest in Condition A as it has freely available score info

Results: Do people copy based on prestige most in Condition C and least in Condition A?

Parameter estimates for choosing prestige

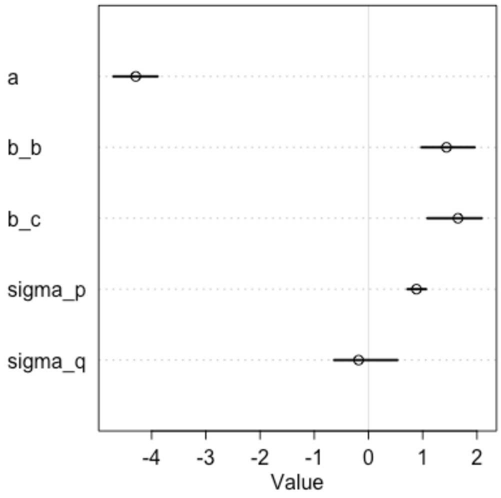

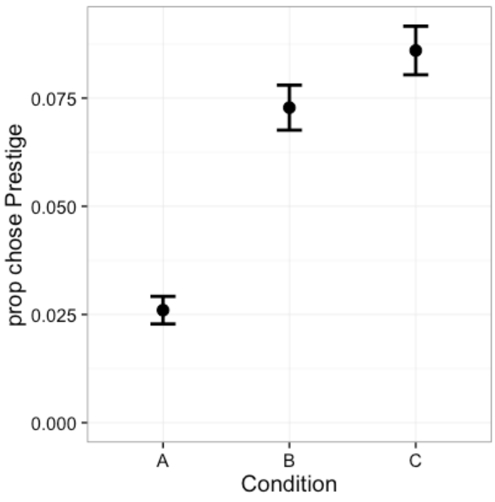

Did participants score highest in Condition A?

Parameter estimates for overall score

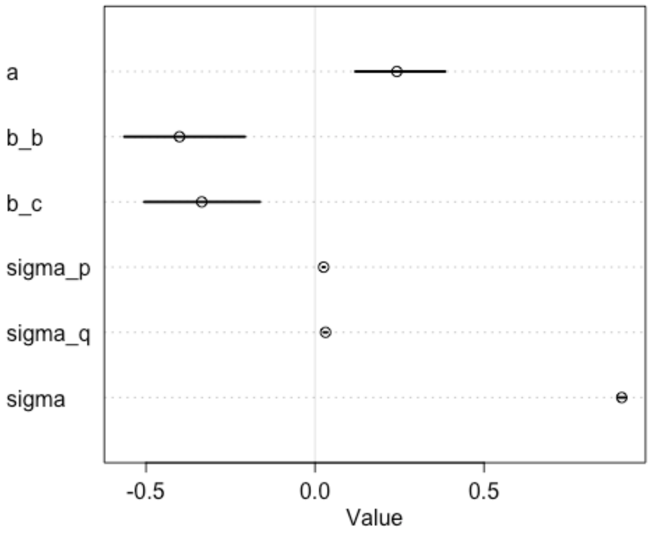

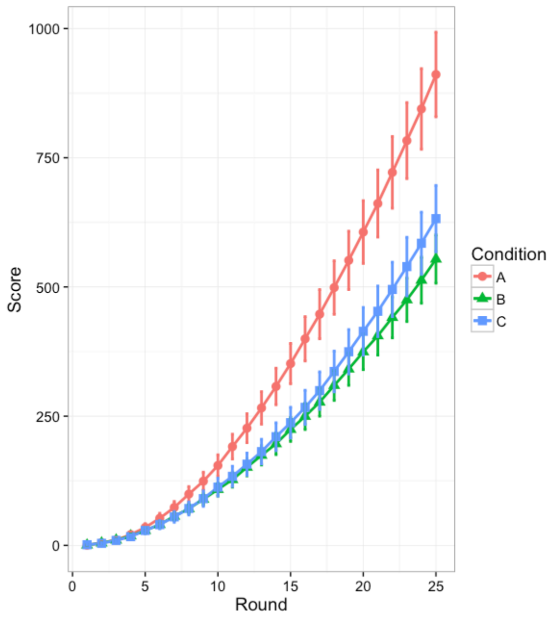

Did they copy more in Condition A and least in Condition C?

Parameter estimates for choosing to copy

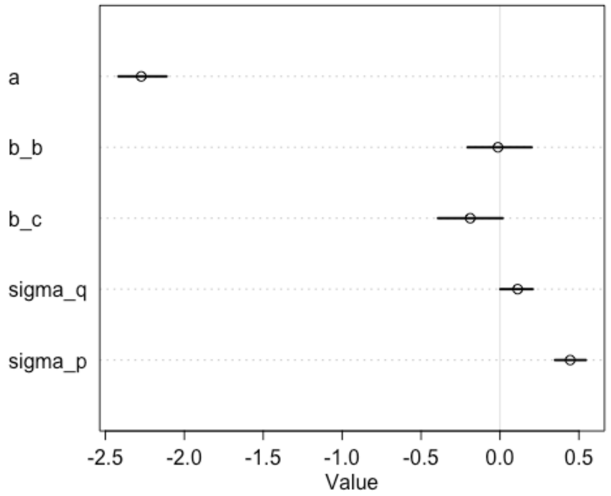

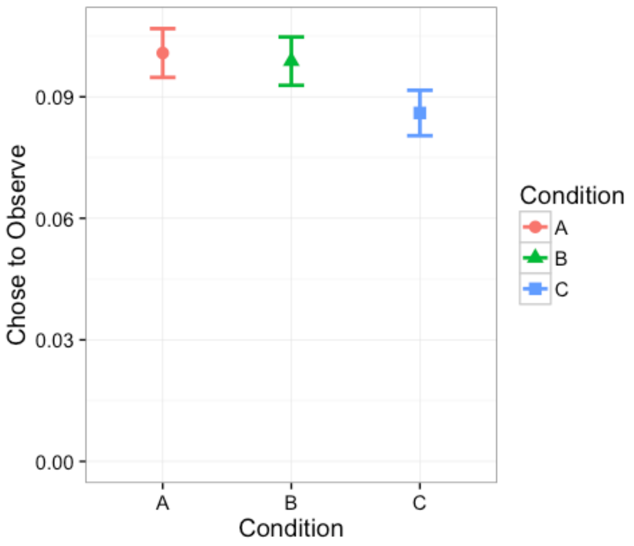

Did they copy the most copied?

Parameter estimates for choosing to copy the most copied

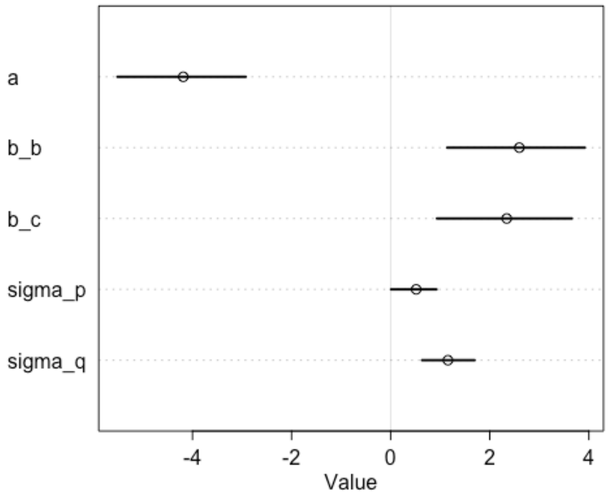

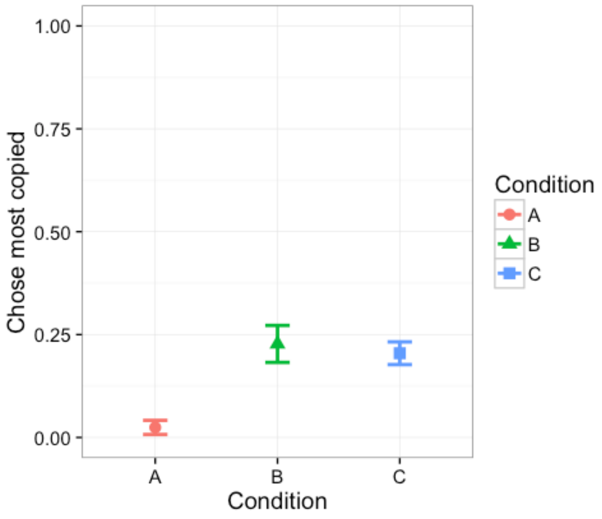

Did they copy the least copied?

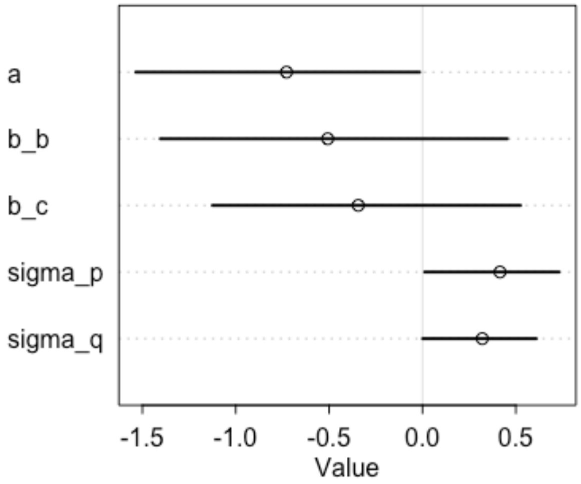

Did they copy the highest scoring?

Parameter estimates for choosing to copy the highest scoring

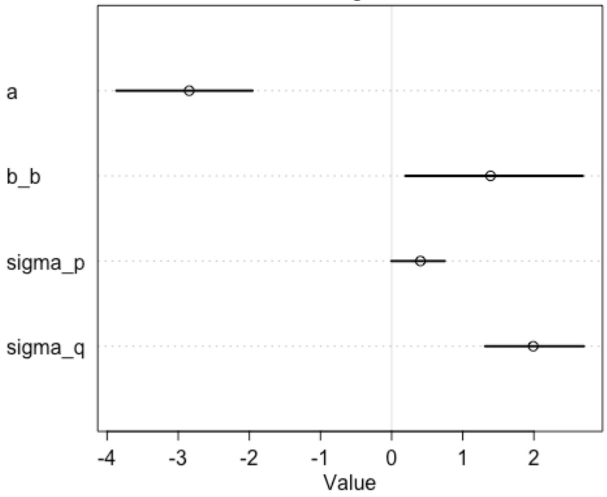

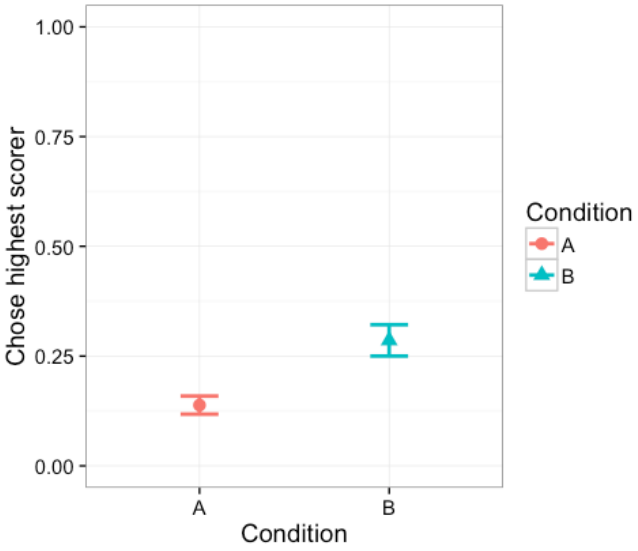

Did they copy the lowest scorer?

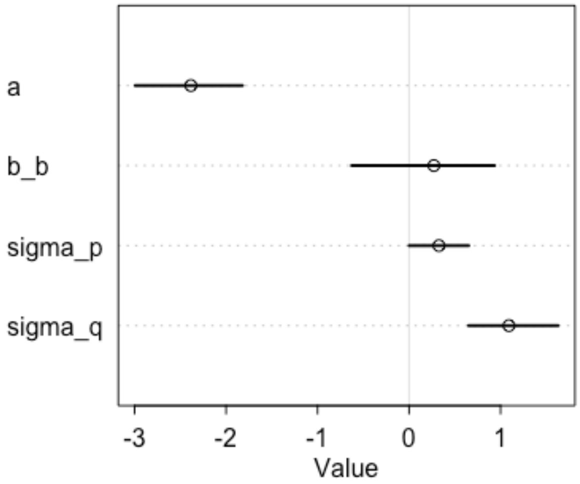

Overall Conclusions

- People will choose to copy based on prestige information when success information is costly or unavailable
- This is more evident on the abstract task, people get distracted by other characteristics in the knowledge-based task (they may think it's useful even though it isn't)
- People don't reliably copy the most copied, but they don't appear to be copying at random either… they avoid copying the least copied.
- People also don't choose to copy the highest scorer available, although they do avoid choosing the lowest scorer available.
- Results should be interpreted with caution: participants reported not wanting to 'copy' in the Qualtrics game, associating it with cheating, and also due to intrinsic enjoyment of quiz, wanting to assess their own individual knowledge. Perhaps incentive for achieving top score was not high enough in the Qualtrics payment scheme to overcome this.

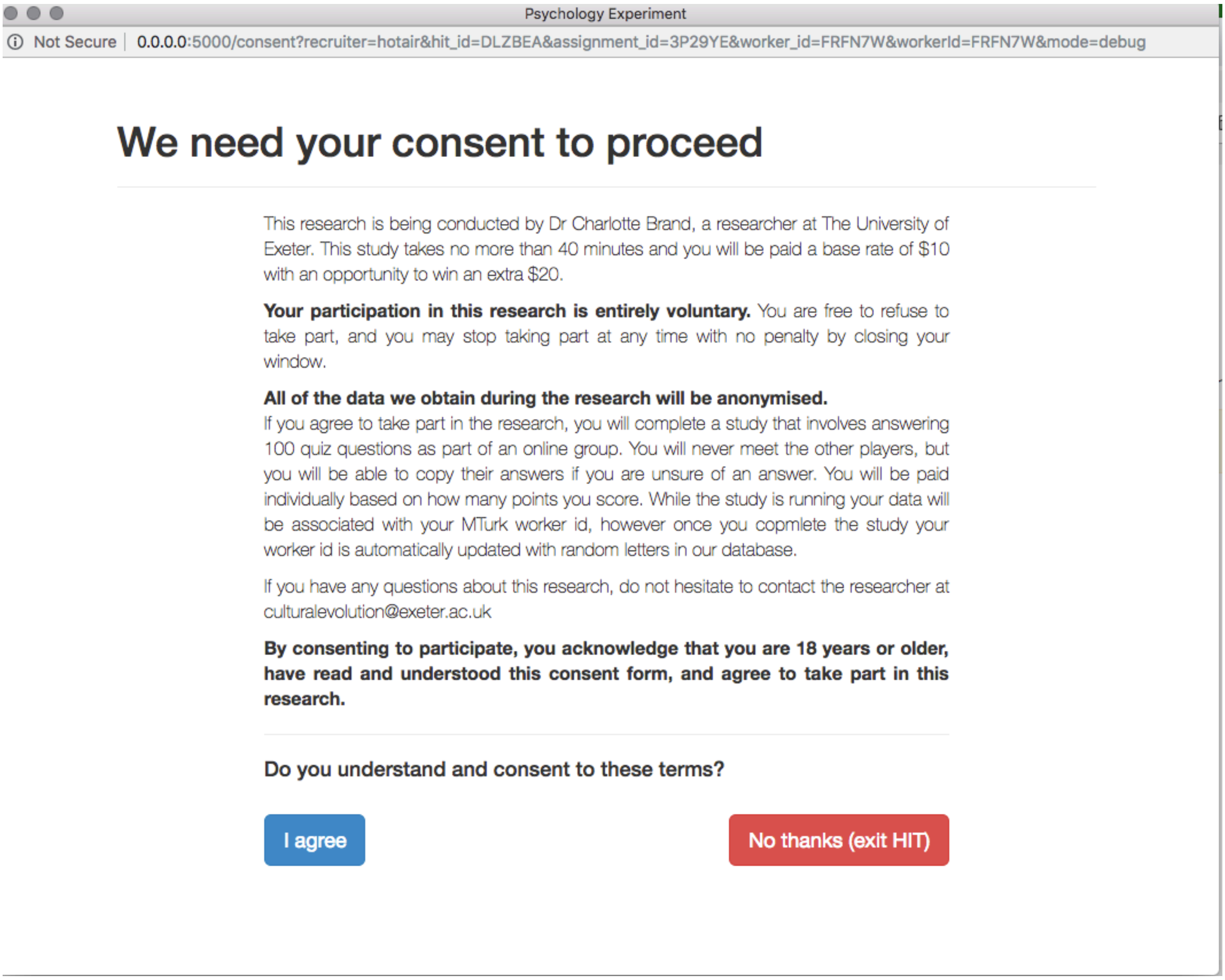
Psychology Experiment

Not Secure | 0.0.0.0:5000/consent?recruiter=hotair&hit_id=DLZBEA&assignment_id=3P29YE&worker_id=FRFN7W&workerId=FRFN7W&mode=debug

We need your consent to proceed

This research is being conducted by Dr Charlotte Brand, a researcher at The University of Exeter. This study takes no more than 40 minutes and you will be paid a base rate of $10 with an opportunity to win an extra $20.

Your participation in this research is entirely voluntary. You are free to refuse to take part, and you may stop taking part at any time with no penalty by closing your window.

All of the data we obtain during the research will be anonymised.
If you agree to take part in the research, you will complete a study that involves answering 100 quiz questions as part of an online group. You will never meet the other players, but you will be able to copy their answers if you are unsure of an answer. You will be paid individually based on how many points you score. While the study is running your data will be associated with your MTurk worker id, however once you copmlete the study your worker id is automatically updated with random letters in our database.

If you have any questions about this research, do not hesitate to contact the researcher at culturalevolution@exeter.ac.uk

By consenting to participate, you acknowledge that you are 18 years or older, have read and understood this consent form, and agree to take part in this research.

Do you understand and consent to these terms?

I agree

No thanks (exit HIT)

Psychology Experiment

Not Secure | 0.0.0.0:5000/instructionsB

Instructions

What you will be doing:

In this experiment, you will be taking part in a quiz with a group of 10-15 other people.
Everyone is given a random Player ID number
You will have to answer **100 general knowledge questions.**
There are four topics: **Geography, Art History, Language recognition, and Weight estimation.**
Some questions have pictures, others do not.
You will have **15 Seconds** to answer each question.
If you do not answer in 15 seconds, you will not score a point for that question.
If you do not respond to a question for over 2 minutes you will delay the rest of your group and therefore will not be able to continue to the end
You will not be told if you answered a question correctly or incorrectly throughout the quiz. You will be told your total score at the end of the quiz.

Points and Payment:

Everyone gets **$10** for completing the quiz. You get one point for each correct answer.
If you score **over 90 points** you will receive **$20** bonus payment.

Asking someone else:

If you are unsure of an answer, you can select **"Ask Someone Else"** to copy the answer of one of the other players.
When you select **"Ask Someone Else"** you will be shown the score of every other player in the group who answered that question.
You will not be able to see their answer, but, **if the player you choose answered correctly, you will also get a point for that question.**
If the person you choose answered incorrectly, you will **not** get a point for that question.

Practice Round:

You will now complete four practice questions before you begin the real quiz, to familiarise yourself with how the buttons work. No one can score points in the practice round.

Begin Practice Round

Practice Round

Question: 'What is the capital city of France?',
Question number:'practice 1',
Round: 0,
Topic: 'Practice',
Wrong Answer: 'Barcelona',
Right Answer: 'Paris',
Picture Question: False,

Question: 'How much does an average chimpanzee weigh?',
Question number:'practice 2',
Round: 0,
Topic: 'Practice',
Wrong Answer: '500kg',
Right Answer: '50kg',
Picture Question: False,

Question: '\"Pepino\" means \"cucumber\" in which language?',
Question number:'practice 3',
Round: 0,
Topic: 'Practice',
Wrong Answer: 'Italian',
Right Answer: 'Spanish',
Picture Question: False,

Question: 'Vincent van Gogh died in which year?',
Question number:'practice 4',
Round: 0,
Topic: 'Practice',
Wrong Answer: '1880',
Right Answer: '1890',
Picture Question: False,

Round 1:

Question: 'In which country is the red dot located?',
Question number:1,
Round: 1,
Topic: 'Geography',
Wrong Answer: 'Guatemala',
Right Answer: 'Belize',
Picture Question: True,

Question: 'In which country is the red dot located?',
Question number:2,
Round:1,

Topic: 'Geography',
Wrong Answer: 'Nigeria',
Right Answer: 'The Ivory Coast',
Picture Question: True,

Question: 'The capital of Hawaii is',
Question number:3,
Round:1,
Topic: 'Geography',
Wrong Answer: 'Waikiki',
Right Answer: 'Honolulu',
Picture Question: False,

Question: 'Saint Helena is an island in',
Question number:4,
Round:1,
Topic:'Georgraphy',
Wrong Answer: 'The Indian Ocean',
Right Answer: 'The South Atlantic Ocean',
Picture Question: False,

Question: 'Which country shares a border with El Salvador?',
Question number:5,
Round:1,
Topic:'Georgraphy',
Wrong Answer: 'Paraguay',
Right Answer: 'Honduras',
Picture Question: False,

Question: 'The capital of the Philippines is?',
Question number:6,
Round:1,
Topic:'Georgraphy',
Wrong Answer: 'Davao',
Right Answer: 'Manila',
Picture Question: False,

Question: 'Which is closest to Finland?',
Question number:7,
Round:1,
Topic:'Georgraphy',
Wrong Answer: 'Moscow',
Right Answer: 'St Petersburg',
Picture Question: False,

Question: 'Which is the largest of The Canary Islands?',
Question number:8,
Round:1,
Topic:'Georgraphy',
Wrong Answer: 'Gran Canaria',
Right Answer: 'Tenerife',
Picture Question: False,

Question: 'Oklahoma state shares a border with?',
Question number:9,
Round:1,
Topic:'Georgraphy',
Wrong Answer: 'Arizona',
Right Answer: 'New Mexico',
Picture Question: False,

Question: 'Amsterdam is nearer to?',
Question number:10,
Round:1,
Topic:'Georgraphy',
Wrong Answer: 'Antwerp',
Right Answer: 'Rotterdam',
Picture Question: False,

Question: 'A pot of nail varnish weighs?',
Question number:11,
Round:1,
Topic:'Weight estimation',
Wrong Answer: '162g',
Right Answer: '62g',
Picture Question: True,

Question: 'A Rubiks cube weighs?',
Question number:12,
Round:1,
Topic: 'Weight',
Wrong Answer: '1.4kg',
Right Answer: '0.14kg',
Picture Question: True,

Question: 'A blue whale weighs?',
Question number:13,

Round:1,
Topic: 'Weight',
Wrong Answer: '1,400kg',
Right Answer: '140,000kg',
Picture Question: False,

Question: 'A tennis ball weighs?',
Question number:14,
Round:1,
Topic: 'Weight',
Wrong Answer: '5.85g',
Right Answer: '58.5g',
Picture Question: False,

Question: 'Which weighs more, on average?',
Question number:15,
Round:1,
Topic: 'Weight',
Wrong Answer: 'A wood pigeon',
Right Answer: 'A seagull',
Picture Question: False,

Question: 'A Boeing 747 (on take-off) weighs?',
Question number:16,
Round: 1,
Topic: 'Weight',
Wrong Answer: '40,000kg',
Right Answer: '400,000kg',
Picture Question: False,

Question: 'A skateboard weighs?',
Question number:17,
Round: 1,
Topic: 'Weight',
Wrong Answer: '34kg',
Right Answer: '3.4kg',
Picture Question: False,

Question: 'A newborn baby weighs?',
Question number:18,
Round: 1,
Topic: 'Weight',
Wrong Answer: '34kg',
Right Answer: '3.5kg',

Picture Question: False,

Question: 'A female giraffe weighs?',
Question number:19,
Round: 1,
Topic: 'Weight',
Wrong Answer: '83kg',
Right Answer: '830kg',
Picture Question: False,

Question: 'Which weighs more?',
Question number:20,
Round: 1,
Topic: 'Weight',
Wrong Answer: 'The London Eye',
Right Answer: 'The Eiffel Tower',
Picture Question: False,

Question: '\"Cal\" means \"horse\" in which language?',
Question number:21,
Round: 1,
Topic: 'Language',
Wrong Answer: 'Hungarian',
Right Answer: 'Romanian',
Picture Question: False,

Question: '\"Kieselstein\" means \"pebbles\" in which language?',
Question number:22,
Round: 1,
Topic: 'Language',
Wrong Answer: 'Russian',
Right Answer: 'German',
Picture Question: False,

Question: '\"Gobierno\" means \"government\" in which language?',
Question number:23,
Round: 1,
Topic: 'Language',
Wrong Answer: 'Portugese',
Right Answer: 'Spanish',
Picture Question: False,

Question: '\"Verre\" means \"glass\" in which language?',

Question number:24,
Round: 1,
Topic: 'Language',
Wrong Answer: 'Italian',
Right Answer: 'French',
Picture Question: False,

Question: '\"Bere\" means \"drink\" in which language?',
Question number:25,
Round: 1,
Topic: 'Language',
Wrong Answer: 'German',
Right Answer: 'Italian',
Picture Question: False,

Question: '\"Kabaha\" means \"shoes\" in which language?',
Question number:26,
Round:1,
Topic: 'Language',
Wrong Answer: 'Swahili',
Right Answer: 'Somali',
Picture Question: False,

Question: '\"Bocadillo\" means \"snack\" in which language?',
Question number:27,
Round:1,
Topic: 'Language',
Wrong Answer: 'French',
Right Answer: 'Spanish',
Picture Question: False,

Question: '\"Rzeka\" means \"river\" in which language?',
Question number:28,
Round: 1,
Topic:'Language',
Wrong Answer: 'Latvian',
Right Answer: 'Polish',
Picture Question: False,

Question: 'The above means \"fire\" in which language?',
Question number:29,
Round:1,
Topic: 'Language',
Wrong Answer: 'Bulgarian',

Right Answer: 'Russian',
Picture Question: True,

Question: 'The above means \"soul\" in which language?',
Question number:30,
Round:1,
Topic: 'Language',
Wrong Answer: 'Armenian',
Right Answer: 'Arabic',
Picture Question: True,

Question: 'The above is an image of a painting by which artist?',
Question number:31,
Round:1,
Topic: 'Art',
Wrong Answer: 'Edouard Manet',
Right Answer: 'Claude Monet',
Picture Question: True,

Question: 'The above is an image of a painting by which artist?',
Question number:32,
Round:1,
Topic: 'Art',
Wrong Answer: 'Rudolf Hausner',
Right Answer: 'Gustav Klimt',
Picture Question: True,

Question: 'Edouard Manet died in',
Question number:33,
Round:1,
Topic: 'Art',
Wrong Answer: '1663',
Right Answer: '1883',
Picture Question: False,

Question: '\"The starry night\" is a famous painting by',
Question number:34,
Round:1,
Topic: 'Art',
Wrong Answer: 'Jackson Pollock',
Right Answer: 'Vincent van Gogh',
Picture Question: False,

Question: '\"The singing butler\" is a painting by the Scottish painter:',
Question number:35,
Round:1,
Topic: 'Art',
Wrong Answer: 'Andrew Geddes',
Right Answer: 'Jack Vettriano',
Picture Question: False,

Question: 'Antoni Gaudi was a Spanish:',
Question number:36,
Round:1,
Topic: 'Art',
Wrong Answer: 'Poet',
Right Answer: 'Architect',
Picture Question: False,

Question: 'Auguste Rodin was a French:',
Question number:37,
Round:1,
Topic: 'Art',
Wrong Answer: 'Architect',
Right Answer: 'Sculptor',
Picture Question: False,

Question: 'Rembrandt was famous for which style of painting?',
Question number:38,
Round:1,
Topic: 'Art',
Wrong Answer: 'Surrealism',
Right Answer: 'Baroque',
Picture Question: False,

Question: 'Paul Gauguin was a famous post-impressionist artist from:',
Question number:39,
Round:1,
Topic: 'Art',
Wrong Answer: 'Italy',
Right Answer: 'France',
Picture Question: False,

Question: 'Edgar Degas famously painted',
Question number:40,
Round:1,
Topic: 'Art',

Wrong Answer: 'water lilies',
Right Answer: 'dancers',
Picture Question: False,

Round 2:

Question: 'In which city is the red dot located?',
Question number:41,
Round:2,
Topic: 'Geography',
Wrong Answer: 'Seattle',
Right Answer: 'Philadelphia',
Picture Question: True,

Question: 'In which country is the red dot located?',
Question number:42,
Round:2,
Topic: 'Geography',
Wrong Answer: 'Greece',
Right Answer: 'Bulgaria',
Picture Question: True,

Question: 'In which country is the red dot located?',
Question number:43,
Round:2,
Topic: 'Geography',
Wrong Answer: 'Laos',
Right Answer: 'Vietnam',
Picture Question: True,

Question: 'Which city is closer to Rome?',
Question number:44,
Round:2,
Topic: 'Geography',
Wrong Answer: 'Florence',
Right Answer: 'Naples',
Picture Question: False,

Question: 'Kazakhstan shares a border with?',
Question number:45,
Round:2,
Topic: 'Geography',
Wrong Answer: 'Tajikistan',
Right Answer: 'Kyrgyzstan',
Picture Question: False,

Question: 'The Virgin Islands are closer to:',
Question number:46,
Round:2,
Topic: 'Geography',
Wrong Answer: 'The Indian Ocean',
Right Answer: 'The Caribbean Sea',
Picture Question: False,

Question: 'Brussels is a city in:',
Question number:47,
Round:2,
Topic: 'Geography',
Wrong Answer: 'Germany',
Right Answer: 'Belgium',
Picture Question: False,

Question: 'Melbourne is a city in:',
Question number:48,
Round:2,
Topic: 'Geography',
Wrong Answer: 'New Zealand',
Right Answer: 'Australia',
Picture Question: False,

Question: 'Paris is closer to:',
Question number:49,
Round:2,
Topic: 'Geography',
Wrong Answer: 'Bordeaux',
Right Answer: 'Calais',
Picture Question: False,

Question: 'Sri Lanka is an island off the coast of:',
Question number:50,
Round:2,
Topic: 'Geography',
Wrong Answer: 'Cambodia',
Right Answer: 'India',
Picture Question: False,

Question: 'Liberia shares a border with:',
Question number:51,
Round:2,

Topic: 'Geography',
Wrong Answer: 'Morocco',
Right Answer: 'Guinea',
Picture Question: False,

Question: 'Oman shares a border with',
Question number:52,
Round:2,
Topic: 'Geography',
Wrong Answer: 'Kuwait',
Right Answer: 'Saudi Arabia',
Picture Question: False,

Question: 'Copenhagen is closer to:',
Question number:53,
Round:2,
Topic: 'Geography',
Wrong Answer: 'Oslo',
Right Answer: 'Gothenburg',
Picture Question: False,

Question: 'The Shetland Islands are part of:',
Question number:54,
Round:2,
Topic: 'Geography',
Wrong Answer: 'Iceland',
Right Answer: 'Scotland',
Picture Question: False,

Question: 'Tokyo is closer to:',
Question number:55,
Round:2,
Topic: 'Geography',
Wrong Answer: 'Kyoto',
Right Answer: 'Hamamatsu',
Picture Question: False,

Question: 'Which dog weighs more, on average?',
Question number:56,
Round:2,
Topic: 'Weight',
Wrong Answer: 'Labrador',
Right Answer: 'Great dane',
Picture Question: False,

Question: 'What is the average weight of a \"pink salmon\"?',
Question number:57,
Round:2,
Topic: 'Weight',
Wrong Answer: '17kg',
Right Answer: '1.7kg',
Picture Question: True,

Question: 'A cricket bat weighs?',
Question number:58,
Round:2,
Topic: 'Weight',
Wrong Answer: '14kg',
Right Answer: '1.4kg',
Picture Question: True,

Question: 'The average weight of a camel is:',
Question number:59,
Round:2,
Topic: 'Weight',
Wrong Answer: '48kg',
Right Answer: '480kg',
Picture Question: True,

Question: 'What does a typical, office fire extinguisher weigh?',
Question number:60,
Round:2,
Topic: 'Weight',
Wrong Answer: '115kg',
Right Answer: '1.15kg',
Picture Question: False,

Question: 'What is the average weight of an oak tree?',
Question number:61,
Round:2,
Topic: 'Weight',
Wrong Answer: '900kg',
Right Answer: '9000kg',
Picture Question: False,

Question: 'What does a typical car tyre weigh?',
Question number:62,

Round:2,
Topic: 'Weight',
Wrong Answer: '0.7kg',
Right Answer: '7kg',
Picture Question: False,

Question: 'Which weighs more, on average?',
Question number:63,
Round:2,
Topic: 'Weight',
Wrong Answer: 'A hen\'s egg',
Right Answer: 'An ostrich egg',
Picture Question: False,

Question: 'Which weighs more, on average?',
Question number:64,
Round:2,
Topic: 'Weight',
Wrong Answer: 'An olive',
Right Answer: 'A fig',
Picture Question: False,

Question: 'How much does an average loaf of bread weigh?',
Question number:65,
Round:2,
Topic: 'Weight',
Wrong Answer: '40g',
Right Answer: '400g',
Picture Question: False,

Question: 'How much does a barrel of whisky weigh?',
Question number:66,
Round:2,
Topic: 'Weight',
Wrong Answer: '2000kg',
Right Answer: '200kg',
Picture Question: False,

Question: 'How much does an average grizzly bear weigh?',
Question number:67,
Round:2,
Topic: 'Weight',
Wrong Answer: '3,400kg',
Right Answer: '340kg',

Picture Question: False,

Question: 'How much does a typical jacket potato weigh?',
Question number:68,
Round:2,
Topic: 'Weight',
Wrong Answer: '18g',
Right Answer: '180g',
Picture Question: False,

Question: 'Which weighs more, on average?',
Question number:69,
Round:2,
Topic: 'Weight',
Wrong Answer: 'An apricot',
Right Answer: 'An avocado',
Picture Question: False,

Question: 'Which weighs more, on average?',
Question number:70,
Round:2,
Topic: 'Weight',
Wrong Answer: 'A coconut',
Right Answer: 'A watermelon',
Picture Question: False,

Question: '\"Chave\" means \"key\" in which language?',
Question number:71,
Round: 2,
Topic:'Language',
Wrong Answer: 'Spanish',
Right Answer: 'Portugese',
Picture Question: False,

Question: '\"Umut\" means \"hope\" in which language?',
Question number:72,
Round: 2,
Topic:'Language',
Wrong Answer: 'German',
Right Answer: 'Turkish',
Picture Question: False,

Question: '\"Nyugodt\" means \"calm\" in which language?',

Question number:73,
Round: 2,
Topic:'Language',
Wrong Answer: 'Polish',
Right Answer: 'Hungarian',
Picture Question: False,

Question: '\"Bloem\" means \"flower\" in which language?',
Question number:74,
Round: 2,
Topic:'Language',
Wrong Answer: 'Danish',
Right Answer: 'Dutch',
Picture Question: False,

Question: '\"Sel\" means \"salt\" in which language?',
Question number:75,
Round: 2,
Topic:'Language',
Wrong Answer: 'German',
Right Answer: 'French',
Picture Question: False,

Question: '\"Schuh\" means \"shoe\" in which language?',
Question number:76,
Round: 2,
Topic:'Language',
Wrong Answer: 'Dutch',
Right Answer: 'German',
Picture Question: False,

Question: '\"Strom\" means \"tree\" in which language?',
Question number:77,
Round: 2,
Topic:'Language',
Wrong Answer: 'Ukranian',
Right Answer: 'Slovak',
Picture Question: False,

Question: '\"Hai\" means \"shark\" in which language?',
Question number:78,
Round: 2,
Topic:'Language',
Wrong Answer: 'Telugu',

Right Answer: 'Finnish',
Picture Question: False,

Question: '\"Dronning\" means \"queen\" in which language?',
Question number:79,
Round: 2,
Topic:'Language',
Wrong Answer: 'Serbian',
Right Answer: 'Norwegian',
Picture Question: False,

Question: '\"Pudel\" means \"bottle\" in which language?',
Question number:80,
Round: 2,
Topic:'Language',
Wrong Answer: 'Bulgarian',
Right Answer: 'Estonian',
Picture Question: False,

Question: '\"Tay\" means \"hand\" in which language?',
Question number:81,
Round: 2,
Topic:'Language',
Wrong Answer: 'Thai',
Right Answer: 'Vietnamese',
Picture Question: False,

Question: '\"Vinaka\" means \"thank you\" in which language?',
Question number:82,
Round: 2,
Topic:'Language',
Wrong Answer: 'Swedish',
Right Answer: 'Fijian',
Picture Question: False,

Question: 'The above means \"to eat\" in which language?',
Question number:83,
Round: 2,
Topic:'Language',
Wrong Answer: 'Swedish',
Right Answer: 'Finnish',
Picture Question: True,

Question: 'The above means \"star\" in which language?',
Question number:84,
Round: 2,
Topic:'Language',
Wrong Answer: 'Macedonian',
Right Answer: 'Greek',
Picture Question: True,

Question: 'The above is \"festival\" written in which language?',
Question number:85,
Round: 2,
Topic:'Language',
Wrong Answer: 'Japanese',
Right Answer: 'Korean',
Picture Question: True,

Question: 'The above is an image of a painting by which artist?',
Question number:86,
Round:2,
Topic: 'Art',
Wrong Answer: 'Franz Kline',
Right Answer: 'Jackson Pollack',
Picture Question: True,

Question: 'The above is an image of a painting by which artist?',
Question number:87,
Round:2,
Topic: 'Art',
Wrong Answer: 'Marcel Duchamp',
Right Answer: 'Salvador Dali',
Picture Question: True,

Question: 'The above is an image associated with which artist?',
Question number:88,
Round:2,
Topic: 'Art',
Wrong Answer: 'Roy Lichtenstein',
Right Answer: 'Andy Warhol',
Picture Question: True,

Question: '\"Flaming June\" is a painting by:',
Question number:89,
Round:2,
Topic: 'Art',

Wrong Answer: 'Jan van Eyck',
Right Answer: 'Sir Frederic Leighton',
Picture Question: False,

Question: '\"The Creation of Adam\" is a painting by:',
Question number:90,
Round:2,
Topic: 'Art',
Wrong Answer: 'Leonardo da Vinci',
Right Answer: 'Michelangelo',
Picture Question: False,

Question: 'Henri Matisse was a French artist of the:',
Question number:91,
Round:2,
Topic: 'Art',
Wrong Answer: '15th Century',
Right Answer: '20th Century',
Picture Question: False,

Question: '\"The Scream\" is a painting by:',
Question number:92,
Round:2,
Topic: 'Art',
Wrong Answer: 'Ernst Ludwig Kirchner',
Right Answer: 'Edvard Munch',
Picture Question: False,

Question: '\"Girl with a Pearl Earring\" is a painting by:',
Question number:93,
Round:2,
Topic: 'Art',
Wrong Answer: 'Peter Paul Rubens',
Right Answer: 'Johannes Vermeer',
Picture Question: False,

Question: '\"The Mona Lisa\" is a painting by:',
Question number:94,
Round:2,
Topic: 'Art',
Wrong Answer: 'Vincent van Gogh',
Right Answer: 'Leonardo da Vinci',
Picture Question: False,

Question: 'Rene Magritte was famous for:',
Question number:95,
Round:2,
Topic: 'Art',
Wrong Answer: 'Impressionism',
Right Answer: 'Surrealism',
Picture Question: False,

Question: 'Frida Kahlo was a famous painter from:',
Question number:96,
Round:2,
Topic: 'Art',
Wrong Answer: 'South Africa',
Right Answer: 'Mexico',
Picture Question: False,

Question: 'Georgia O\'Keefe was a famous painter of the:',
Question number:97,
Round:2,
Topic: 'Art',
Wrong Answer: '16th Century',
Right Answer: '20th Century',
Picture Question: False,

Question: 'Edward Hopper painted \"Nighthawks\" in:',
Question number:98,
Round:2,
Topic: 'Art',
Wrong Answer: '1742',
Right Answer: '1942',
Picture Question: False,

Question: '\"The Birth of Venus\" is a painting by:',
Question number:99,
Round:2,
Topic: 'Art',
Wrong Answer: 'Leonardo da Vinci',
Right Answer: 'Sandro Boticelli',
Picture Question: False,

Question: 'James Abbott Mcneil Whistler painted \"Whistler\'s Mother\" in:',
Question number:100,
Round:2,

Topic: 'Art',
Wrong Answer: '1571',
Right Answer: '1871',
Picture Question: False,
